# Clinical Features, Video Head Impulse Test, and Subjective Visual Vertical of Acute and Symptom-Free Phases in Patients with Definite Vestibular Migraine

**DOI:** 10.3390/biomedicines13040825

**Published:** 2025-03-30

**Authors:** Franko Batinović, Davor Sunara, Nikolina Pleić, Vana Košta, Jelena Gulišija, Ivan Paladin, Zrinka Hrgović, Mirko Maglica, Zoran Đogaš

**Affiliations:** 1 Department of Otorhinolaryngology, University Hospital of Split, Spinčićeva 1, 21000 Split, Croatia; davor.sunara@gmail.com (D.S.); ivan.paladin@gmail.com (I.P.); mirko.maglica@gmail.com (M.M.); 2 Department of Biology and Human Genetics, School of Medicine, University of Split, Šoltanska 2A, 21000 Split, Croatia; 3 Department of Neurology, University Hospital of Split, Spinčićeva 1, 21000 Split, Croatia; vanakosta@gmail.com (V.K.); jelena.gulisija.neura@gmail.com (J.G.); 4 Department of Family Medicine, Health Center of Split—Dalmatia County, 21000 Split, Croatia; zrinkahrgovic@hotmail.com; 5 Department of Neuroscience and Sleep Medicine Center, School of Medicine, University of Split, Šoltanska 2A, 21000 Split, Croatia; zdogas@gmail.com

**Keywords:** vestibular migraine, vertigo, vestibular tests, video head impulse test, subjective visual vertical

## Abstract

**Background/Objectives**: The most frequent neurologic cause of recurrent vertigo is vestibular migraine (VM). However, its diagnosis relies primarily on patients’ histories, as specific diagnostic tests for VM are currently lacking. We aimed to examine and compare clinical features, vestibulo-ocular reflexes (VORs), and subjective visual vertical (SVV) between the ictal (IC) and inter-ictal (II) phases in VM patients. **Methods**: A repeated-measures study involved 31 patients with definite VM. Vestibular function was assessed using a video head impulse test (vHIT) to evaluate VOR results, and SVV testing to determine verticality perception. Otoneurological examination, including migraine-related disability, was noted. Analyses of repeated measures for numerical traits (SVV deviations, VOR, and clinical outcomes) were conducted using a linear mixed model (LMM), with phase, age, and sex as fixed effects and individual-specific random intercepts. Differences between the IC and II phases for dichotomous variables were analyzed using the χ2 or Fisher’s exact test. **Results**: The LMM analysis revealed that SVV deviations were significantly higher ictally (IC-ly) (β = 0.678, *p* = 1.51 × 10^−6^) than interictally (II-ly). VOR results remained normal across phases (*p* > 0.05), and refixation saccades did not differ significantly based on vHIT results (*p* > 0.05). Nausea (100% vs. 38.71%, *p* = 6.591 × 10^−8^), photophobia (100% vs. 35.48%, *p* = 1.839 × 10^−8^), and phonophobia (90.32% vs. 6.45%, *p* = 9.336 × 10^−12^) were significantly more frequent IC-ly than II-ly. **Conclusions**: Our findings highlight phase-dependent alterations in spatial orientation, with increased SVV deviations IC-ly despite stable VOR. The significant differences in migraine-associated symptoms reinforce the dynamic nature of VM. These results emphasize the importance of timing in vestibular assessments and suggest that SVV testing during IC VM episodes may enhance diagnostic accuracy.

## 1. Introduction

Vestibular migraine (VM) is the most common neurologic cause of episodic vertigo [1,2] with a prevalence of 1–3% in the adult population [3,4]. Although it is less common than migraines, which affect 13–16% of the adult population in Croatia and worldwide [5], VM has received increasing interest in the last two decades but remains under-recognized [6].

The cause of VM remains a matter of speculation [7]. The underlying pathophysiological mechanisms of vertigo, headache, and sensory hyperexcitability in patients with VM involve both peripheral and central vestibular pathways [8]. Vestibular, visual, and somatosensory information shapes the brain’s perception of visual verticality [9]. The central vestibulo-cerebellar connection is vital for processing spatial orientation and upright perception [8,9], while the cerebellar Purkinje cells of the flocculus and paraflocculus are essential for vestibulo-ocular reflexes (VORs) [10]. The reciprocal connection between the vestibular and trigeminal caudal nuclei facilitates interaction between vestibular stimulation and migraine headaches and is currently regarded as the primary mechanism underlying VM [7,11].

Diagnostic criteria for definite VM (dVM) have been represented jointly by the International Headache Society and the Bárány Society [12,13]. The challenges in diagnosing VM come from its broad spectrum of vestibular symptoms and the lack of a specific diagnostic test [6,11]. The key to diagnosis is a meticulous history of migraine and vestibular symptoms as well as a detailed otoneurological examination during the VM attacks [6,12].

No specific diagnostic abnormality in otoneurological examinations can be measured in a VM, neither in the ictal (IC) nor the inter-ictal (II) periods [6]. The IC phase refers to the period during an acute attack when symptoms such as vertigo, dizziness, nausea, and imbalance are present. The II phase is the symptom-free period between attacks, during which patients may still experience subtle vestibular abnormalities, motion sensitivity, or imbalance, even in the absence of a full-blown episode [14]. The results of diagnostic tests in VM patients tend to vary significantly [6,7]. During the IC phase of VM, some patients have central spontaneous nystagmus, central positional nystagmus, or a combination of the two [15]. In most VM patients, the overall otoneurologic examination remains normal interictally (II-ly) [16]. Objective vestibular and radiological tests have assisted in excluding other otoneurological disorders [2,16].

Despite its high prevalence and significant impact on quality of life and healthcare costs, VM remains under-diagnosed [11]. Only 8 to 20% of VM patients receive an accurate diagnosis during their initial visit [3], primarily due to inadequate history-taking and a lack of targeted diagnostic testing [11].

In recent clinical studies, measuring the VOR results with a video head impulse test (vHIT) [17,18], and the degree of the subjective visual vertical (SVV) represent a helpful enhancement in diagnosing VM patients [8,19,20]. The vHIT is anatomically linked to the function of the semicircular canals in the peripheral vestibular system, brainstem motor nuclei, and extraocular muscles. It detects vestibular hypofunction by measuring gain reduction and identifying covert or overt saccades. Compared to the bed head impulse test (bHIT), vHIT offers faster, objective results with greater sensitivity, as it can detect covert saccades even in the presence of central compensation—findings that may go unnoticed with bHIT [21]. SVV is used to assess abnormalities in perceived vertical orientation. In healthy individuals, the perception of verticality is highly accurate and relies on sensory input from the visual, vestibular, and somatosensory systems. The otolithic organs within the vestibular system, specifically the utricle and saccule, play a key role in detecting gravity and maintaining vertical perception. Dysfunction in otoliths or the nerve pathways transmitting vestibular signals to the brain can disrupt this perception, leading to a noticeable tilt in one’s sense of verticality [22].

Our study aimed to find the optimal combination of otoneurological tests that can hasten and improve the diagnosis of VM, facilitating quicker diagnosis and treatment while reducing the disease burden.

To date, no one has compared the results of clinical examinations, VOR, and SVV measurements in the IC and II phases among the same dVM patients. Our study, therefore, will examine and compare the clinical otoneurological results and the vestibulo-ocular diagnostic peculiarities measured by vHIT and SVV in both IC and II phases in dVM patients.

## 2. Materials and Methods

### 2.1. Study Design

**Figure 1:**
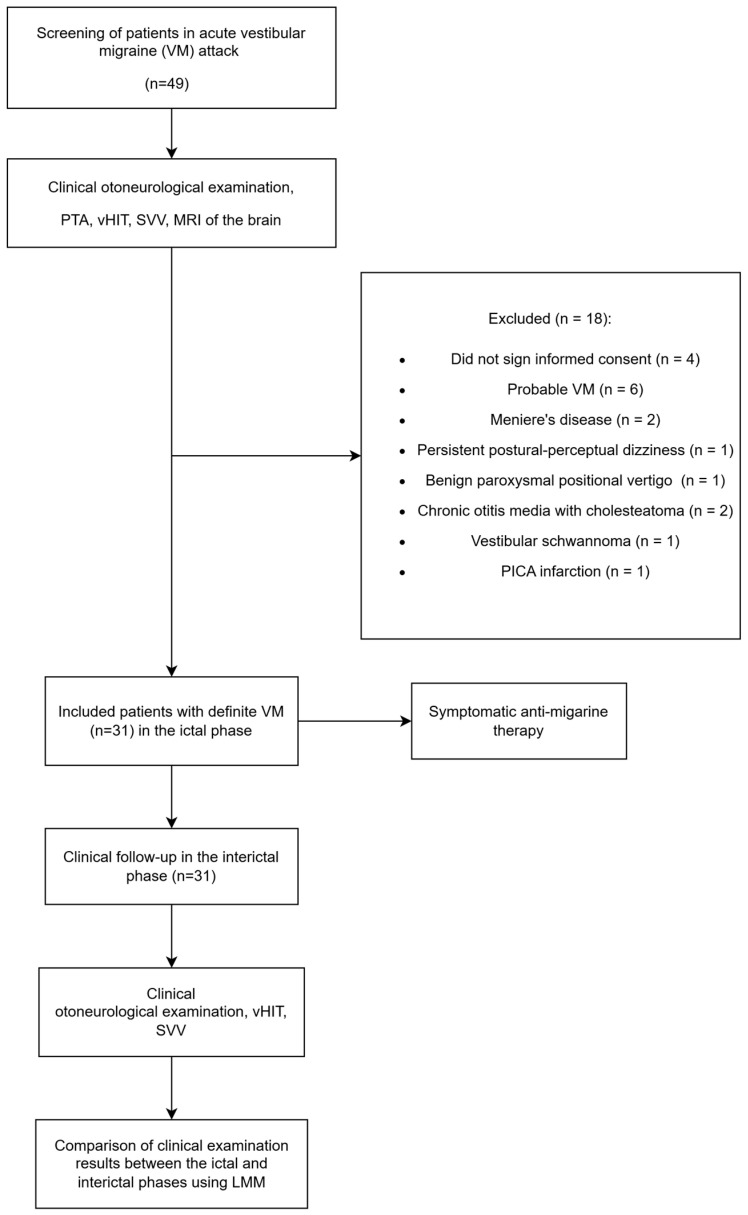
Flowchart of dVM patients’ initial screening and follow-up examinations.

This repeated-measures study was conducted at the Ear, Nose, and Throat (ENT) tertiary clinic at the University Hospital of Split, Croatia, between May 2022 and May 2024. Ethical approval for this study was granted by the Ethical Committee of the University Hospital of Split on 6 September 2021 (No. 2181-147-01/06/M.S.-21-02). Our research adhered to the STROBE checklist (Supplementary File S1) and was pre-registered at Open Science Framework (https://archive.org/details/osf-registrations-ef7ng-v1, accessed on 24 November 2024). All adult VM patients who presented at the Emergency ENT Department (EED) during an acute VM attack were examined by an otorhinolaryngologist and a neurologist during the initial IC phase and later in the II phase (Figure 1).

### 2.2. Participants

Forty-nine VM patients arrived at the EED. Finally, thirty-one adult patients were included and provided written informed consent (Supplementary File S2). They also met the criteria for dVM as defined by the International Classification of Headache Disorders (ICHD) and the Bárány Society agreement (Supplementary File S3). We excluded (i) patients who arrived at the EED ≥ 24 h after an acute VM attack; (ii) patients with confirmed other audio-vestibular and neurological disorders; (iii) patients with functional dizziness; and (iv) patients with ocular issues (cataracts, strabismus, eye surgery, etc.).

Examinations were conducted through two clinical visits. The first visit represents the IC phase of VM. Within 24 h of admission, all enrolled patients underwent a structured clinical examination conducted by an ENT specialist and a neurologist. This examination included a detailed medical history, otoneurologic evaluation, recording of anthropometric parameters, measuring the vHIT and SVV, pure tone audiometry (PTA), completion of the numeric rating scale (NRS) for vertigo and headache intensity, and brain magnetic resonance imaging (MRI). All patients received rescue therapy for acute VM attacks, which included *granisetron* (1 mg in 250 mL saline as intravenous infusion) and *metamizol* (2.5 g in 100 mL saline as intravenous infusion). This treatment is part of the standard therapy in our ENT clinic for patients experiencing acute VM attacks. Nineteen patients did not take any central nervous system-acting medications prior to their VM attacks. Six patients took *benzodiazepines* and two took *dimenhydrinate*. Three patients were on *selective serotonin reuptake inhibitors*, and one was using *topiramate* for chronic therapy.

The second visit represented the II phase and served as a follow-up examination conducted between the fifth and tenth day after the IC phase of VM. This follow-up was conducted at the ENT tertiary clinic at the University Hospital of Split, Croatia.

The same ENT specialist and neurologist confirmed that dVM patients were in the II phase based on the follow-up otoneurological examination. Patients underwent the same assessments as in the IC phase, including vHIT, SVV assessment, and NRS scoring for vertigo and headache intensity. No patients were lost to follow-up; however, two patients had missing vHIT data during the follow-up (interictal) assessment, and one of them also had missing vHIT data in the ictal phase. All other patients had complete data.

### 2.3. Otoneurological Examination

The acute dVM was confirmed by an otorhinolaryngologist and a neurologist with over five years of experience in their subspecialties. A clinical otoneurological examination included the following: otoscopy, the HINTS plus battery (head impulse test, nystagmus, Skew test, hearing loss), the STANDING algorithm (augmentation of HINTS plus battery with positional nystagmus and gait testing), an examination of the ocular motor system (the range of eye movements, saccades, smooth pursuit, ocular lateropulsion, ocular tilt reaction, and head-shaking), an assessment of spontaneous nystagmus (in primary gaze, gaze holding, and with Frenzel’s glasses) and positional testing (Dix–Hallpike and lateral roll tests) [6,23]. We also rated truncal ataxia, House–Brackmann (facial nerve function), and ABCD2 scores (arterial blood pressure, clinical features of stroke, duration of symptoms, and diabetes) [24]. We evaluated the patient’s subjective perception of the intensity of vertigo and headache using the 11-point NRS during the IC and II phases of VM. The scales were classified as follows: 0–3, mild; 4–6, moderate; and 7–10, severe vertigo or headache. PTA was objectified IC-ly to assess hearing levels to distinguish between VM, sudden hearing loss, and Meniere’s disease [11]. A neuroradiologist evaluated all VM patients using a 1.5 Tesla brain MRI (Siemens, Erlangen, Germany)

### 2.4. Specific Diagnostic Tools

#### 2.4.1. Video Head Impulse Test (vHIT)

All dVM patients were examined by the same otorhinolaryngologist using a 3-dimensional video head impulse test (vHIT; GN Otometrics, Taastrup, Denmark 2019) in the IC and II VM phases. The vHIT represents a reliable testing battery for the differential diagnosis of acute vestibulopathy and vestibular insult in the EED [25,26]. It evaluates the gain and dysfunction of the VOR in each inner ear semicircular canal (SC) [25,27]. Reduced or increased VOR gain and evident refixation saccades are the two pathological signs observed in vHIT [25,28]. The lightweight goggles, equipped with an integrated video-oculography camera with a sampling rate of 220 Hz, were tightly fixed to the patient’s head. The patients were instructed to fixate their eyes on a dot on the wall at about a 1.2 m distance. They needed to follow the dot in each direction to achieve calibration. After calibration, an experienced otorhinolaryngologist, who had conducted over a thousand vHITs, advised patients to relax their necks before testing each SC using quick and unpredictable head movements, at velocities ranging from 150 to 200°/s. Thirty impulses were delivered per each semicircular canal. The vHIT manufacturer (ICS otometric) defines normal VOR gain limits as between 0.8 and 1.2 for lateral canals or 0.7 and 1.2 for vertical canals [25,29]. Refixation saccades can be categorized as cover (occurring during a head movement) or overt (occurring after a head movement) [30,31].

#### 2.4.2. Subjective Visual Vertical (SVV)

The same otorhinolaryngologist examined the function of the otolith organs using SVV IC-ly and II-ly. SVV is a reliable method for evaluating the function of central vestibular pathways and the perception of upright posture [19]. Patients without otoneurological disorders, when sitting upright at rest, have an SVV within a range of ±2.5° [19]. We used a measurement technique with a mobile phone SVV (Apple, Cupertino, USA) application (app) described by Day T 2020 [32]. The visual vertical (VV) iPhone app is an effective and user-friendly method for measuring the SVV [32]. The patients were seated upright in a completely dark room, looking into an opaque plastic bucket equipped with the active VV iPhone app and positioning their heads about 60 cm away from the rim. The VV iPhone app at the bottom of the bucket features a straight red line (length 10 cm, width 2 mm) with a zero line at 90° corresponding to the true vertical. The patients adjusted the bucket at eye level and the red line to what they perceived as the gravitational vertical. The test was repeated six times, and the otorhinolaryngologist calculated the average value of the VV iPhone app results, representing the final SVV score [19,20].

### 2.5. Statistical Analysis

The data are presented as absolute and relative frequencies for categorical variables and as mean ± standard deviation (SD) or median (lower quartile–upper quartile) for continuous variables in Table 1, Table 2 and Table 3.

A repeated-measures analysis of the SVV, vHIT, and clinical parameters in observed patients was performed using a linear mixed model (LMM) in which the mentioned parameters were a dependent variable and time was modelled as a fixed effect (ictal phase vs. inter-ictal phase). Sex (coded as 1 = male, 2 = female) and age were included as additional covariates in the LMM. A random intercept was included in each LMM using individual identification (ID) to account for individual-specific variability, allowing each patient to have a unique baseline measurement. This approach captured intra-individual correlations in repeated measures, improving model accuracy and preventing bias in the estimation of fixed effects. Unlike ANOVA, which requires complete data across all time points and would otherwise exclude participants with partial data, the LMM approach utilizes all available data points, preserving the sample size, modelling individual differences and complex temporal effects more flexibly, and providing greater reliability and validity in repeated-measures analyses by including data from all 31 patients.

The difference in the distribution of dichotomous variables between the IC and II phases was tested using Fisher’s exact test.

The level of statistical significance was set to 0.05. Statistical analyses were performed using the statistical programming language R version 4.3.0 [33]. Figure 1 presents the study design and analysis flowchart.

## 3. Results

A total of 49 patients were initially recruited from the tertiary clinic. However, four patients did not provide informed consent during their first visit in the IC phase and were subsequently excluded from further analyses. As a result, the final study cohort consisted of 45 patients who completed assessments during both the IC and II phases.

Additionally, 14 patients were excluded due to specific diagnoses that did not meet the inclusion criteria. Six patients were excluded because of a diagnosis of acute probable VM, as the analysis was restricted to cases of acute definite VM. One patient was excluded due to persistent postural-perceptual dizziness alongside acute VM, and another because of benign paroxysmal positional vertigo (BPPV) along with acute VM. Two patients were excluded because of Ménière’s disease, and two due to chronic otitis media with cholesteatoma. Further exclusions comprised one patient with a posterior inferior cerebellar artery (PICA) infarction, and another with a vestibular schwannoma impacting the left vestibular nerve. After these exclusions, 31 patients with dVM remained for final analysis.

The demographic and clinical characteristics of dVM patients are presented in Table 1. The mean age of the patients was 46.6 years (SD = 15.9). The gender distribution was skewed, with 28 women (90.32%) and a smaller proportion of men. The duration of the most recent VM attack was reported to be 10 h (Q1 = 6, Q3 = 12), and the number of VM attacks reported up to the point of assessment was 10 (Q1 = 10, Q3 = 15). Notably, acute VM attacks predominantly occurred in the morning, with most cases reported around 8 AM. Regarding lifestyle habits, 10 dVM patients (32.26%) reported coffee consumption, while 21 patients (67.74%) did not. No dVM patients (0%) reported alcohol use, and 11 patients (35.48%) reported smoking, while 20 patients (64.52%) did not smoke. In terms of marital status, seven dVM patients (22.58%) were single, eighteen patients (58.06%) were married, four patients (12.90%) were divorced, two patients (6.45%) were widowed, and no patients (0%) were separated.

Table 2 shows the clinical characteristics of dVM patients during the IC phase of dVM. During this phase, 83.87% of patients experienced spontaneous vertigo, 9.68% had positional vertigo, and 6.45% experienced both types. Vomiting occurred in 10 patients (32.26%) during the IC phase, but it was not assessed II-ly. In terms of triggers, the most commonlyreported ones were poor sleep (64.52%), weather change (58.06%), and stress (48.39%). Other triggers included sharp head movements (22.58%), excessive computer use (12.90%), excessive cell phone use (9.68%), intense light and sound (9.68%) or spontaneous onset (32.26%).

In terms of the timing of migraine headaches and VM attacks, most patients (93.55%) reported experiencing the headache prior to the VM attack, while 6.45% noted it occurring during the VM attack, and 3.23% experienced it afterward.

The presence of aura was reported by 41.94% of dVM patients, all of whom experienced it before the VM attack. None of the dVM patients reported aura occurring during or after the VM attack, while 58.06% did not experience an aura.

Over half of the dVM patients (58.06%) had no noticeable hearing symptoms. Among those who reported auditory disturbances, tonal tinnitus was the most prevalent symptom, affecting 12.90% unilaterally and 16.13% bilaterally. A sense of ear fullness was reported by 6.45% of dVM patients. No cases of unilateral or bilateral hearing loss were found.

Regarding other clinical symptoms, phobic symptoms were present in 32.26% of dVM patients. However, none reported experiencing diplopia, dysphagia, or dysphonia (0%). A family history of migraine was prevalent, with 90.32% of patients reporting at least one affected family member.

In the otoneurological examination, all dVM patients showed inconspicuous findings on otoscopy. Based on the ABCD2 score, the majority (80.65%) scored 1, while 19.35% scored 2. None of the dVM patients had a score of 3 or higher. All patients (100%) exhibited mild truncal ataxia (grade 1), with no cases of moderate (grade 2) or severe (grade 3) ataxia. Similarly, all dVM patients had a House–Brackmann facial expression score of 1/6, indicating normal facial nerve function.

In the PTA assessment, the median hearing threshold was 16 dB (10–22.50 dB) in the right ear and 15 dB (11–22.50 dB) for the left ear, indicating no significant hearing impairment among dVM patients.

The severity of vertigo, assessed using the NRS, had a median score of 8 (7–9), indicating a high intensity of vertigo IC-ly. In contrast, headache severity was lower II-ly, with a median NRS score of 2 (1–3).

The MRI analysis revealed UBO lesions in 48.39% of dVM patients, predominantly in the deep white matter, as well as in the frontal, subcortical, and temporal-parietal regions. Most dVM patients exhibited bilateral lesions, particularly in the frontal and parietal cortical areas. In contrast, 51.61% of dVM patients showed no detectable UBO lesions on MRI.

The findings presented in Table 3 summarize the comparison of symptoms and clinical signs between the IC and II phases in dVM patients.

During the IC phase, all dVM patients (100%) experienced nausea, while in the II phase, nausea was reported by 38.71% of patients. This difference was statistically significant (*p* = 6.591 × 10^−8^). Similarly, photophobia was present in all dVM patients (100%) during the IC phase but decreased to 35.48% in the II phase, showing a significant reduction (*p* = 1.839 × 10^−8^). Phonophobia was reported by 90.32% of dVM patients during the IC phase, but only 6.45% in the II phase, indicating a highly significant difference (*p* = 9.336 × 10^−12^). In contrast, osmophobia remained relatively stable between the IC (9.68%) and II (3.22%) phases, with no statistically significant difference (*p* = 0.612).

Regarding vestibular and neurological assessments, the bHIT, Skew test (cover-uncover test), saccade, and head shaking test were negative in all dVM patients across both phases, making statistical comparisons not applicable (N/A).

For smooth pursuit, abnormalities were observed in 80.65% of dVM patients during the IC phase and 90.32% in the II phase; the difference was not statistically significant (*p* = 0.473). Similarly, the Romberg test was positive in 6.45% of patients during the IC phase but negative in all dVM patients II-ly, with no significant difference (*p* = 0.492).

However, a significant difference was found in spontaneous nystagmus, which was observed in 22.58% of dVM patients during the IC phase but was absent II-ly (*p* = 0.011), indicating a notable reduction in this clinical sign. Additionally, nystagmus in the lateral roll and Dix–Hallpike positional tests were observed in 29.03% of dVM patients during the IC phase, decreasing to 6.45% II-ly, showing a statistically significant reduction (*p* = 0.043). However, the lateral roll test alone showed no positive cases in either phase, making statistical comparison not applicable (N/A).

**Figure 2:**
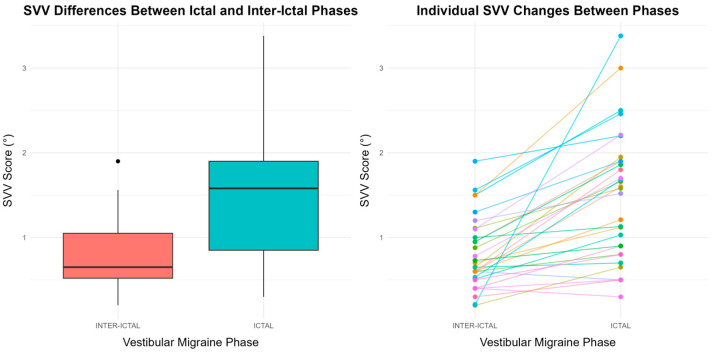
Boxplot and spaghetti plot illustrating subjective visual vertical (SVV) differences between ictal and inter-ictal phases of vestibular migraine. (**Left**) The boxplot displays the distribution of SVV scores across both phases, showing the median, interquartile range, and outliers. (**Right**) The spaghetti plot depicts individual SVV changes between the two phases, demonstrating inter-individual variability. Distinct line colors are used to represent individual participants.

The linear mixed model (LMM) results indicate that SVV significantly differed between IC and II VM periods (β = 0.678, SE = 0.113, *p* = 1.51 × 10^−^⁶), with SVV deviations being significantly higher during IC VM episodes (Figure 2). This finding suggests a significant phase-dependent alteration in spatial orientation perception in dVM patients. In contrast, no significant effects of gender (*p* = 0.463) or age (*p* = 0.557) were observed on SVV (Supplementary File S4, Table S1).

**Figure 3:**
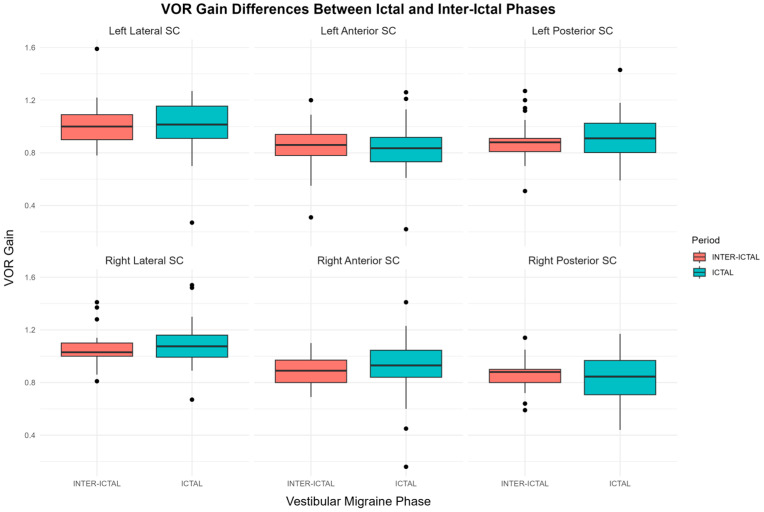
Boxplots comparing vestibulo-ocular reflex (VOR) results across the six semicircular canals (SC) during ictal and inter-ictal phases of vestibular migraine. The first row represents left ear semicircular canals (lateral, anterior, and posterior SC), while the second row represents right ear semicircular canals. Each boxplot displays the median, interquartile range, and outliers for VOR results in both conditions.

For vestibular function, none of the VOR results (for lateral, anterior, or posterior SCs) in either ear exhibited significant phase-dependent changes between the IC and II phases of dVM (Table 3, *p* > 0.05 and Figure 3). Similarly, gender and age did not significantly influence VOR function, suggesting that vestibular responses remain stable across VM phases and are not modulated by demographic factors (Supplementary File S4, Tables S2–S7). The analysis of vHIT refixation saccades showed no significant difference between the IC and II phases of dVM. Overt saccades were observed in 15.05% of all tested SCs during the IC phase and 11.83% during the II VM phase (*p* = 0.396, Table 3). Covert saccades were noted in 11.83% of SCs during the IC phase and 9.14% in the II phase of dVM (*p* = 0.423, Table 3).

A comprehensive presentation of all statistical results, including detailed tables, box plots, interaction plots, and spaghetti plots illustrating the effects of phases (IC vs. II) and gender on SVV and vestibular function, is provided in Supplementary File S4.

## 4. Discussion

Our study demonstrated significant phase-dependent alterations in spatial orientation perception in dVM patients, as evidenced by higher SVV deviations during the IC phase compared to the II phase. In contrast, the VOR function, assessed through vHIT, remained stable across both phases, with no significant changes in results for all SCs. Additionally, we observed no significant influence of gender or age on SVV or vHIT results. These findings suggest that while spatial orientation is notably impaired during acute VM attacks, peripheral vestibular function remains largely unaffected, reinforcing the hypothesis of central vestibular involvement in VM pathophysiology.

The findings from previous studies on the duration of VM attacks have shown considerable inconsistency. For instance, Neuhauser et al. observed that the duration of vertigo experienced by VM patients could vary significantly, lasting anywhere from just a few minutes to several days [34]. Notably, they found that over 50% of VM patients reported experiencing vertigo for more than one hour [3,34]. These results align with our observations. Similarly, the frequency of attacks varies widely among VM patients, with different authors reporting a broad range of frequencies [27,35]. Consistent with the findings of Liu et al., our study also observed that acute VM attacks predominantly occurred during the morning hours [36]. Furthermore, patients tended to experience more severe VM episodes after waking up [36]. This pattern suggests a potential chronobiological influence on VM attacks, warranting further investigation into the underlying mechanisms that may contribute to the temporal distribution and severity of symptoms.

The onset of vestibular symptoms follows a sex-specific pattern, typically occurring during the perimenopausal period in women and men’s third decade of life [1,6]. Notably, VM predominantly affects women, with reported prevalence ratios reaching up to 5:1 compared to men [6,14,34,35]. These sociodemographic findings are consistent with our data.

Regarding triggers of VM attacks, Beh et al. identified stress as the most common precipitating factor, followed by bright lights, sleep deprivation, and weather changes [6]. Among behavioral triggers in VM patients, the most frequently reported included skipping meals and activities involving excessive head movements, such as household cleaning or frequently shifting focus between computer screens and paper documents, as well as physical exercise [6]. Our findings largely align with these results. However, in contrast to previous findings, where bright lights and loud sounds were among the most frequent triggers, our study identified them as the least common precipitants of VM attacks.

Previous studies have consistently reported characteristic symptoms during the IC phase of VM, with spontaneous vertigo occurring in more than 50% of patients and positional vertigo reported in approximately 10–20%, depending on the study [6,16,34,37,38,39]. The temporal relationship between headache and vestibular symptoms in VM is highly variable [40]. Headaches may or may not accompany VM episodes, with studies showing that between 24% and 75% of patients consistently experience headaches during VM attacks [34]. Additionally, headaches associated with VM are often less severe than the patient’s typical migraine headaches [34]. In our study, the vast majority of dVM patients (93.55%) experienced headaches before the onset of a VM attack, while 6.45% reported headaches occurring during the VM attack, and 3.23% experienced headaches afterwards. It is important to highlight a key finding from Beh’s study, which emphasizes that vertigo is the predominant symptom in VM [6]. At the same time, headaches are typically less severe and are often overshadowed by vertigo’s intensity [6]. This aligns with our findings, as dVM patients predominantly emphasized a higher intensity of vertigo IC-ly, usually paying minimal attention to headaches or not reporting them as a primary concern. Compared to previous studies, phonophobia, photophobia, and nausea were reported more frequently by our participants, with over 90% experiencing these symptoms [6,34,37,41]. Conversely, osmophobia was present in approximately 10% of dVM patients during the IC phase, a finding that is in complete agreement with previous research [6]. One of the key strengths of our study lies in the comparative analysis of symptom frequency between the IC and II phases of VM. Our findings demonstrate that symptoms such as nausea, photophobia, phonophobia, and vomiting were significantly more prevalent during the IC phase, highlighting their strong association with acute VM attacks. In contrast, osmophobia did not show a significant difference in occurrence between the two phases, suggesting that its presence may not be as closely linked to the IC phase of dVM as other symptoms. This distinction underscores the importance of understanding symptom variability across different phases of dVM, which may have implications for diagnosis and management.

Testing SVV primarily evaluates the vestibular system’s role in perceiving verticality [42]. A meta-analysis by Obrero-Gaitán et al. found that patients with common primary headaches, such as tension-type headaches and migraines, exhibited more significant alterations in SVV test results [43]. These findings are consistent with those of Miller et al., who reported that patients with migraines and VM demonstrated a wider range of vertical deviation in SVV assessments [44]. We found that dVM patients exhibited significantly greater SVV deviations during IC episodes, indicating a phase-dependent alteration in spatial orientation perception. However, it is essential to note that despite these variations, SVV values remained within the reference range in both phases. To the best of our knowledge, only Winnick et al. have previously conducted SVV testing using a mobile phone app alongside vHIT in the same study, which included 27 VM patients [8]. Their findings indicated that VM patients exhibited normal vHIT results, and normal SVV values in the upright head position [8]. In contrast to them, our study specifically included dVM patients. We examined all six SC canals and compared VOR and SVV results IC-ly and II-ly. Most importantly, we found significantly higher SVV results in dVM patients IC-ly than II-ly.

However, Winnick et al. found a significant difference between VM patients and healthy controls during static head tilts of 20° to the right, with VM patients showing greater SVV deviations [8]. These findings suggest that VM patients may be sensitive to displacements in the roll plane, and their tendency to overestimate the tilt position could contribute to more substantial errors in upright perception.

Furthermore, unlike previous studies [8,44], we analyzed the impact of age and gender on SVV deviations during both IC and II VM phases. We found no significant association between these factors and SVV alterations. These results reinforce the idea that alterations in spatial orientation are primarily related to the disease process rather than demographic factors. A key strength of our study lies in the comparative analysis of patients with confirmed dVM during both symptomatic and asymptomatic phases. This approach highlights the potential utility of SVV as a primary diagnostic tool for VM—an entity that remains inadequately recognized and frequently underdiagnosed [1]. Abnormal SVV deviations in dVM patients may be associated with a dysfunction in the “higher-order” multisensory integration responsible for spatial orientation, specifically involving vestibular and somatosensory inputs that encode head, neck, and eye positions [8].

Abnormalities in vHIT among dVM patients are reported to be rare, with reduced lateral canal VOR gain ranging from 2.2% to 18% across studies [28,45]. However, some reports describe increased gain in vertical SCs, attributed to vestibular hypersensitivity in VM patients [35]. During the IC phase, low gains in all SCs, though not statistically significant, have previously been observed, along with an increased frequency of covert saccades [27]. Young et al. found that most VM patients have normal vHIT results II-ly [46]. In contrast to previous studies, we compared vHIT results in both VM phases and examined all SCs. However, we found no significant difference between VOR results in either ear. Additionally, gender and age did not significantly influence VOR function, suggesting that vestibular responses remain stable across both VM phases. Moreover, we noted the presence of refixation saccades, with no significant difference in their incidence between the IC and II VM phases. In line with our study, refixation saccades were absent, and VOR gain in the vHIT was normal in 73% of VM patients in the study by ElSherif et al. [31]. Also, Kang et al. reported that only 11% of VM patients exhibited abnormal VOR results but did not note the presence of refixation saccades. This implies that VM patients generally have an intact VOR reflex arc [17]. On the contrary, according to other clinical studies, half of the VM patients on vHIT had refixation saccades IC-ly, suggesting peripheral vestibular involvement [27].

In patients with VM who do not exhibit vestibular or ocular motor dysfunction, the observed errors in upright perception may be attributed to neural processes localized within the cerebral hemispheres, which are known to play a significant role in spatial orientation [9]. The “higher order” dysfunction aligns with our study, in which VOR results were mainly normal in dVM patients. This proposed mechanism corresponds with the broader role of multisensory integration in the pathophysiology of migraines [47].

The main limitation of our study is that it was conducted at a single tertiary center, which, along with a potentially limited sample size, may affect the generalizability of our results.

The primary strengths of our study lie in its novelty and comprehensive approach. We are the first to conduct a detailed otoneurological examination, including vHIT and SVV testing, in patients with dVM diagnosed according to Bárány Society and ICHD criteria, during both IC and II phases. The dVM patients were examined by the same otorhinolaryngologist and neurologist within 24 h from the onset of VM attack. vHIT was performed by an experienced otorhinolaryngologist with thirty impulses for each SC. Also, measuring SVV with a mobile phone app is quick and user-friendly and can be easily used in an emergency ambulance. We believe that comparing the same dVM patients IC-ly and II-ly has the best potential to find specific diagnostic tests for diagnosing dVM. Additionally, no previous studies have evaluated the influence of age and gender on SVV and vHIT across these phases, making our findings particularly significant. The comparison of the overt and covert saccades amplitudes in vHIT in IC and II VM periods needs to be addressed in future studies, as well as testing SVV with a head tilt for the same patients IC-ly and II-ly.

## 5. Conclusions

Patient’s anamnesis remains the cornerstone and gold standard for accurately diagnosing dVM. Physical examination, including vHIT and SVV results during both symptomatic and asymptomatic phases, serves as a valuable diagnostic tool. When combined with other vestibular and neurological assessments, these tests enhance a more comprehensive evaluation, improving the recognition and accuracy of dVM diagnosis. By integrating objective measures with clinical assessment, our study emphasizes the importance of a multidimensional diagnostic approach, ultimately paving the way for better identification, management, and treatment of this underdiagnosed condition.

## Figures and Tables

**Table 1:** Demographic, clinical, and lifestyle characteristics of dVM patients.

	IC and II Phase (n = 31)
**Age** (years)	46.6 (15.9)
**Female**	28 (90.32%)
**Duration of the last VM attack**	10 (6–12)
**Nr. of VM attacks until now**	10 (10–15)
**Marital status**	
Single	7 (22.58%)
Married	18 (58.06%)
Divorced	4 (12.90%)
Widowed	2 (6.45%)
Separated	0 (0%)
**Coffee use**	
Yes	10 (32.26%)
No	21 (67.74%)
**Alcohol use**	
Yes	0 (0%)
No	31 (100%)
**Smoking**	
Yes	11 (35.48%)
No	20 (64.52%)

Variables are represented as mean (SD), median (Q1–Q3), or absolute frequency (relative frequency). dVM, definite vestibular migraine; IC, ictal phase; II, inter-ictal phase.

**Table 2:** Clinical and otoneurological characteristics of dVM patients during the IC phase of VM.

	IC Phase (n = 31)
**Type of vertigo**	
Spontaneous	26 (83.87%)
Positional	3 (9.68%)
Both spontaneous and positional	2 (6.45%)
**Vomiting**	
Yes	10 (32.26%)
No	21 (67.74%)
**Trigger**	
Poor sleep	20 (64.52%)
Weather change	18 (58.06%)
Stress	15 (48.39%)
Spontaneous start	10 (32.26%)
Sharp movement of the head	7 (22.58%)
Excessive computer use	4 (12.90%)
Excessive cell phone use	3 (9.68%)
Strong light and sound	3 (9.68%)
**Nr. of current vestibular symptoms**	
2	1 (3.23%)
3	9 (29.03%)
4	21 (67.74%)
**Migraine headache**	
No	0 (0%)
Before the VM attack	29 (93.55%)
During the VM attack	2 (6.45%)
After the VM attack	1 (3.23%)
**Aura**	
No	18 (58.06%)
Before the VM attack	13 (41.94%)
During the VM attack	0 (0%)
After the VM attack	0 (0%)
**Hearing**	
Inconspicuous	18 (58.06%)
Tonal tinnitus unilaterally	4 (12.90%)
Tonal tinnitus bilaterally	5 (16.13%)
Sense of fulness	2 (6.45%)
Hearing loss unilaterally	0 (0%)
Hearing loss bilaterally	0 (0%)
**Phobic symptoms**	
Yes	10 (32.26%)
No	21 (67.74%)
**Diplopia, dysphagia, dysphonia**	
Yes	0 (0%)
No	31 (100%)
**Migraine in family**	
Yes	28 (90.32%)
No	3 (9.68%)
**Otoneurological examination**	
**OTS**	
Inconspicuous/negative	31 (100%)
Pathologic/positive	0 (0%)
**ABCD2**	
1	25 (80.65%)
2	6 (19.35%)
**Truncal ataxy**	
1	31 (100%)
**House–Brackmann score**	
1/6	31 (100%)
**PTA right (dB)**	16 (10–22.50)
**PTA left (dB)**	15 (11–22.50)
**NRS for vertigo**	8 (7–9)
**NRS for headache**	2 (1–3)
**MRI (UBO lesion)**	15 (48.39%)

Variables are represented as mean (SD), median (Q1–Q3), or absolute frequency (relative frequency). ABCD2, arterial blood pressure, clinical features of stroke, duration of symptoms, diabetes; dVM, definite vestibular migraine; IC, ictal phase;; MRI, magnetic resonance imaging; NRS, numerical rating scale; OTS, otoscopy; PTA, pure tone audiometry.

**Table 3:** Comparison of symptoms between ictal and inter-ictal phases of dVM patients.

	IC Phase (n = 31)	II Phase (n = 31)	*p*-Value
**Nausea**			**6.591 × 10^−8 a^**
Yes	31 (100%)	12 (38.71%)	
No	0 (0%)	19 (61.29%)	
**Photophobia**			**1.839 × 10^−8 a^**
Yes	31 (100%)	11 (35.48%)	
No	0 (0%)	20 (64.52%)	
**Phonophobia**			**9.336 × 10^−12 a^**
Yes	28 (90.32%)	2 (6.45%)	
No	3 (9.68%)	28 (90.32%)	
**Osmophobia**			0.612 ^a^
Yes	3 (9.68%)	1 (3.22%)	
No	28 (90.32%)	30 (96.77%)	
**bHIT**			N/A
Inconspicuous/negative	31 (100%)	31 (100%)	
Pathologic/positive	0 (0%)	0 (0%)	
**Skew test**			N/A
Inconspicuous/negative	31 (100%)	31 (100%)	
Pathologic/positive	0 (0%)	0 (0%)	
**Smooth pursuit**			0.473 ^a^
Inconspicuous/negative	6 (19.35%)	3 (9.68%)	
Pathologic/positive	25 (80.65%)	28 (90.32%)	
**Saccade**			N/A
Inconspicuous/negative	31 (100%)	31 (100%)	
Pathologic/positive	0 (0%)	0 (0%)	
**Head shaking test**			N/A
Inconspicuous/negative	31 (100%)	31 (100%)	
Pathologic/positive	0 (0%)	0 (0%)	
**Romberg test**			0.492 ^a^
Inconspicuous/negative	29 (93.55%)	31 (100%)	
Pathologic	2 (6.45%)	0 (0%)	
**Spontaneous nystagmus**			**0.011 ^a^**
Inconspicuous/negative	24 (77.42%)	31 (100%)	
Pathologic/positive	7 (22.58%)	0 (0%)	
**Nystagmus in Dix–Hallpike test**			**0.043 ^a^**
Inconspicuous/negative	22 (70.97%)	29 (93.55%)	
Pathologic/positive	9 (29.03%)	2 (6.45%)	
**Nystagmus in Lateral roll test**			
Inconspicuous/negative	31 (100%)	31 (100%)	N/A
Pathologic/positive	0 (0%)	0 (0%)	
**SVV**	1.58 (0.85–1.9)	0.65 (0.52–1.5)	**1.51 × 10^−6 b^**
**Existence of refixation saccades and VOR in vHIT**			
Overt saccades	28 (15.05%)	22 (11.83%)	0.396 ^c^
Covert saccades	22 (11.83%)	17 (9.14%)	0.423 ^c^
**Right ear**			
Lateral SC	1.09 (0.175)	1.06 (0.127)	0.083 ^b^
Anterior SC	0.912 (0.231)	0.896 (0.120)	0.723 ^b^
Posterior SC	0.846 (0.174)	0.87 (0.116)	0.416 ^b^
**Left ear**			
Lateral SC	1.01 (0.205)	1.01 (0.162)	0.915 ^b^
Anterior SC	0.836 (0.197)	0.846 (0.166)	0.770 ^b^
Posterior SC	0.909 (0.186)	0.892 (0.159)	0.729 ^b^

Variables are represented as mean (SD), median (Q1–Q3), or absolute frequency (relative frequency). The presence of saccades was calculated across all 6 semicircular canals. Statistically significant p-values are marked in bold. bHIT, bed head impulse test; IC, ictal phase, II, inter-ictal phase; SC, semicircular canal; SVV, subjective visual vertical; vHIT, video head impulse test; VOR, vestibulo-ocular reflex. ^a^ Fisher’s exact test, ^b^ Linear mixed model, ^c^ χ2 test.

## Data Availability

The data presented in this study are available upon reasonable request from the corresponding author.

## Supplementary Materials

The following supporting information can be downloaded at: https://www.mdpi.com/article/10.3390/biomedicines13040825/s1, Supplementary File S1—STROBE checklist; Supplementary File S2—Informed consent form; Supplementary File S3—Diagnostic criteria for dVM; Supplementary File S4—Results of the LMM. References [12,13] are cited in the Supplementary Materials.
