# Supplementary material for: Clinical Features, Video Head Impulse Test, and Subjective Visual Vertical of Acute and Symptom-Free Phases in Patients with Definite Vestibular Migraine
Source: Biomedicines. 2025 Mar 30;13(4):825. doi: 10.3390/biomedicines13040825 (PMC12024932; doi:10.3390/biomedicines13040825)
Supplement: Supplementary file 1 [file biomedicines-13-00825-s001.zip › Supplementary File S1 - STROBE checklist.pdf]

Supplementary File S1

STROBE Statement—Checklist of items that should be included in reports of ***cross-sectional studies***

MAIN RESEARCHERS AND INSTITUTIONS:
This study is conducted within the doctoral dissertation of Franko Batinović, MD, Department of Otorhinolaryngology, University Hospital of Split, Spinčićeva 1, 21000 Split, Croatia.
Phone: +385-7-719-5505; E-mail: fbatinovic1@gmail.com
The principal investigator is Prof. Zoran Đogaš, MD, PhD, Department of Neuroscience and Sleep Medicine Center, School of Medicine, University of Split, Šoltanska 2, 21 000 Split, Croatia
Phone: +385-21-557-905; E-mail: zdogas@mefst.hr
Corresponding author: Nikolina Pleić, PhD, Department of Biology and Human Genetics, School of Medicine, University of Split, Šoltanska 2, 21000 Split, Croatia. Email: npleic@mefst.hr

	**Item No**	**Recommendation**	**Line/ Page No.**
**Title and abstract**	1	(*a*) Indicate the study's design with a commonly used term in the title or the abstract	Lines 1-4, Page 1
		(*b*) Provide in the abstract an informative and balanced summary of what was done and what was found	Lines 23-44, Page 1
**Introduction**			
Background/rationale	2	Explain the scientific background and rationale for the investigation being reported	Lines 47-82, Page 2
Objectives	3	State specific objectives, including any prespecified hypotheses	Lines 83-86, Page 2
**Methods**			
Study design	4	Present key elements of study design early in the paper	Lines 90-99, Page 3, Figure 1
Setting	5	Describe the setting, locations, and relevant dates, including periods of recruitment, exposure, follow-up, and data collection	Lines 104-129, Pages 4-5
Participants	6	(*a*) Give the eligibility criteria, and the sources and methods of selection of participants	Lines 104-129, Pages 4-5
Variables	7	Clearly define all outcomes, exposures, predictors, potential confounders, and effect modifiers. Give diagnostic criteria, if applicable	Lines 130-205, Pages 5-6
Data sources/ measurement	8*	For each variable of interest, give sources of data and details of methods of assessment (measurement). Describe comparability of assessment methods if there is more than one group	Lines 130-205, Pages 5-6

Bias	9	Describe any efforts to address potential sources of bias	Lines 184-205, Page 6
Study size	10	Explain how the study size was arrived at	Sample size justification for VM patients: We calculated the sample size for two proportions using Epitools software (https://epitools.ausvet.com.au/samplesize) with a confidence level of 0.95, a desired power of 0.8, and a two-tailed test. We entered the values 0.20 (in the previous two years, about 20% of all patients with vertigo who came in the Emergency Otorhinolaryngology Department in Split had VM) and 0.50 (approximately 50% of VM patients complained of anxiety problems) and calculated that we should have 44 VM patients in a two year study.
Quantitative variables	11	Explain how quantitative variables were handled in the analyses. If applicable, describe which groupings were chosen and why	Lines 184-205, Page 6
Statistical methods	12	(*a*) Describe all statistical methods, including those used to control for confounding	Lines 184-205, Page 6
		(*b*) Describe any methods used to examine subgroups and interactions	Lines 184-205, Page 6
		(*c*) Explain how missing data were addressed	Lines 184-205, Page 6
		(*d*) If applicable, describe analytical methods taking account of sampling strategy	Lines 184-205, Page 6
		(*e*) Describe any sensitivity analyses	Lines 184-205, Page 6
**Results**			
Participants	13*	(a) Report numbers of individuals at each stage of study—eg numbers potentially eligible, examined for eligibility, confirmed eligible, included in the study, completing follow-up, and analysed	Lines 207-219, Pages 6-7
		(b) Give reasons for non-participation at each stage	Lines 207-219, Pages 6-7
		(c) Consider use of a flow diagram	Figure 1
Descriptive data	14*	(a) Give characteristics of study participants (eg demographic, clinical, social) and information on exposures and potential confounders	Lines 220-231, Page 7, Table 1
		(b) Indicate number of participants with missing data for each variable of interest	Table 1
Outcome data	15*	Report numbers of outcome events or summary measures	Table 1, 2 and 3

Main results	16	(*a*) Give unadjusted estimates and, if applicable, confounder-adjusted estimates and their precision (eg, 95% confidence interval). Make clear which confounders were adjusted for and why they were included	Table 1, 2 and 3
		(*b*) Report category boundaries when continuous variables were categorized	Table 1, 2 and 3
		(*c*) If relevant, consider translating estimates of relative risk into absolute risk for a meaningful time period	Table 1, 2 and 3
Other analyses	17	Report other analyses done—eg analyses of subgroups and interactions, and sensitivity analyses	Lines 347-349, Page 13
**Discussion**			
Key results	18	Summarise key results with reference to study objectives	Lines 351-358, Page 13
Limitations	19	Discuss limitations of the study, taking into account sources of potential bias or imprecision. Discuss both direction and magnitude of any potential bias	Lines 466-467, Page 16
Interpretation	20	Give a cautious overall interpretation of results considering objectives, limitations, multiplicity of analyses, results from similar studies, and other relevant evidence	Lines 359-466, Pages 13-16
Generalisability	21	Discuss the generalisability (external validity) of the study results	Lines 359-466, Pages 13-16
**Other information**			
Funding	22	Give the source of funding and the role of the funders for the present study and, if applicable, for the original study on which the present article is based	Line 498, Page 16

*Give information separately for exposed and unexposed groups.

Note: An Explanation and Elaboration article discusses each checklist item and gives methodological background and published examples of transparent reporting. The STROBE checklist is best used in conjunction with this article (freely available on the Web sites of PLoS Medicine at http://www.plosmedicine.org/, Annals of Internal Medicine at http://www.annals.org/, and Epidemiology at http://www.epidem.com/). Information on the STROBE Initiative is available at www.strobe-statement.org.
