# Supplementary material for: Clinical Features, Video Head Impulse Test, and Subjective Visual Vertical of Acute and Symptom-Free Phases in Patients with Definite Vestibular Migraine
Source: Biomedicines. 2025 Mar 30;13(4):825. doi: 10.3390/biomedicines13040825 (PMC12024932; doi:10.3390/biomedicines13040825)
Supplement: Supplementary file 1 [file biomedicines-13-00825-s001.zip › Supplementary File S2 - Informed consent.pdf]

Supplementary File S2

The text of the informed consent form for respondents who expressed a willingness to participate.

MAIN RESEARCHERS AND INSTITUTIONS:

This study is conducted within a doctoral dissertation of Franko Batinović, MD, Department of Otorhinolaryngology, University Hospital of Split, Spinčićeva 1, 21000 Split, Croatia.
Phone: +385-7-719-5505; E-mail: fbatinovic1@gmail.com

The principal investigator is Prof. Zoran Đogaš, MD, PhD, Department of Neuroscience and Sleep Medicine Center, School of Medicine, University of Split, Šoltanska 2, 21 000 Split, Croatia.
Phone: +385-21-557-905; E-mail: zdogas@mefst.hr

Corresponding author: Franko Batinović, MD, Department of Otorhinolaryngology, University Hospital of Split, Spinčićeva 1, 21000 Split, Croatia.
Phone: +385-7-719-5505; E-mail: fbatinovic1@gmail.com

INFORMED CONSENT TO PARTICIPATE IN THE RESEARCH

DURATION OF THE RESEARCH: 24 months.

PLACE OF STUDIES:

- Department of Ear, Nose, and Throat with Head and Neck Surgery, Clinical Hospital Center Split, Spinčićeva 1, 21000 Split, Croatia
- Clinical Department of Diagnostic and Interventional Radiology, Clinical Hospital Center Split, Spinčićeva 1, 21000 Split, Croatia

INVITATION:

Dear respondents, we invite you to participate in a scientific study investigating oto-neurological specifics and meteorological triggers in patients with vestibular migraine (VM). We will analyze your clinical features in the anamnesis and oto-neurological status in the acute and asymptomatic phases of VM. This notice will provide you with information designed to help you decide if you want to participate in this scientific research. Before you decide, we want you to understand why this research is being conducted and what it involves. Therefore, please read this notice carefully. If you do not understand some of the words in this notice, you can ask the doctors and members of the research team involved in this scientific research about them.

PREVIOUS KNOWLEDGE ABOUT THIS PROBLEM:

Migraine is the primary headache that affects 18 % of women and 6 % of men worldwide, with the highest incidence between the ages of 25 and 44. Migraine is considered the third most common disorder, and the seventh most common cause of disability in the world. The prevalence of migraine in Croatia is 16 %, in Europe 15 %, and ranges from 12 % to 27.5 %, depending on the country. It is most often divided into migraine without aura (classic migraine) and migraine with aura. The newer classification of migraines (from 2012 and 2018) introduces a new entity - VM, which is the most common episodic vertigo in neurology and occurs in 1 % of the world's adult population. The most common triggers of VM are stress, sleep deprivation, strong light, noise, and weather changes, and there is a common genetic predisposition in the background. Classic migraine in most cases occurs in the morning from 8 to 12 o'clock with symptoms of pulsating unilateral headache accompanied by nausea and sensitivity to light, noise, and odors. Patients with VM have identical symptoms with a prominent symptom of vertigo, which can last from 5 minutes to 72 hours and greatly impair the quality of life of able-bodied individuals. Medicines for the acute attack and prevention of VM are identical to medicines for classic migraine, but patients often refuse to use them. Patients with VM have poorer quality of life and psychiatric comorbidities. Numerous clinical studies have analyzed the anamnestic and oto-neurological specificities in patients with VM. However, only one established a positive correlation between the occurrence of a sudden change in meteorological parameters (temperature and air pressure) and an acute attack of VM.

This is the first study to examine in more detail in the acute and symptom-free period of VM in the same patients the vestibular-ocular reflex for all six semicircular canals of the balance center via the *video head impulse test* (vHIT). We would also examine the *subjective visual vertical* (SVV) in an acute attack of VM and compare it with the results in the symptom-free phase of the disorder. Also, our goal is to answer why patients refuse to use the prescribed preventive therapy for VM. In conclusion, a more detailed analysis of clinical symptoms, meteorological triggers, quality of life, and reasons for not using prescribed preventive antimigraine therapy can be an important basis for a better understanding of VM.

THE HYPOTHESES OF OUR RESEARCH ARE:

1. The vHIT results will show an increased gain of the vestibulo-ocular reflex (VOR) above 1.2 in more than 50 % of patients with acute VM attack, and a normal result (0.8 to 1.2) in more than 80 % of patients in the symptom-free phase of the VM.
2. SVV will be in the reference range (0 ° to 2,5 °) in the acute and symptom-free phase of VM in more than 50 % of patients.

THE GOALS OF OUR STUDIES:

Examination of clinical oto-neurological specifics in acute and symptom-free phases of VM. The ultimate goals of our research are to examine clinical oto-neurological specifics, vestibular-ocular reflexes, and SVV in acute and symptom-free phases of VM patients.

YOUR ROLE OF RESPONDENTS IN THIS SCIENTIFIC RESEARCH:

You will be applied to the diagnostic tests described below during this research. These diagnostic tests and procedures are commonly used in medical and research settings. The Dalmatian subject will visit the researcher at the EED, Clinical Hospital Center (CHC) Split, where he will be familiarized with all procedures that will be performed during the 24-hour examination and the next clinical visit in five to ten days. You will become acquainted with the research's contents and conditions, as well as the personnel who will conduct the research. The entire procedure of the first clinical visit (in an acute attack of the disorder) takes between 24 and 72 hours. It entails taking a thorough history, an oto-neurological clinical examination by an ENT and neurology specialist (DS and VK), filling out questionnaires, and an examination of the auditory and vestibular systems. At the second clinical visit, in the symptom-free phase of the disorder, we will record magnetic resonance imaging (MRI) of the brain and repeat the oto-neurological status, filling the NRS scale for the intensity of vertigo and headache and examining the balance centers. These searches are chronologically arranged and elaborated in detail below.

THE FOLLOWING PROCEDURES WILL BE PERFORMED DURING THIS RESEARCH:

1. Taking anamnestic data and examination of respondents: Specialists in ENT and neurology with more than 5 years of specialist work experience at the CHC Split will take comprehensive anamnestic data from you and perform a detailed clinical examination. A specialist in neurology and ENT will perform an oto-neurological examination, which includes: a local examination of the ear (otoscopy), examination of the function of the seventh cerebral nerve (facial expression tests); examination of vestibular-ocular reflex (bulbomotor with emphasis on spontaneous, provocative and positional nystagmus, smooth following and saccades, positional tests, cover test, bad head impulse test, head-shaking test), vestibulospinal and vestibulocerebellar reflex arc (walking tests, walking, anticipatory tests, Romberg test, etc.). This procedure will be repeated at a follow-up examination in 5 to 10 days, usually lasting up to 45 minutes.
2. Recording of anthropometric parameters, exact time of arrival, and place of residence: Your body weight will be measured on a diagnostic scale, and an altimeter will be used to determine body height to obtain body mass index (BMI). We perform this test only in the acute phase of the disorder and it will last from 5 to 15 minutes. Due to the analysis of meteorological parameters, we will record the exact place of residence of the patient and the exact time of arrival at the reception.
3. Recording of the vHIT and SVV:
   The vHIT test is a computerized examination of the function of the VOR arc. The search is possible in the emergency service, and we do not need more than twenty minutes to record the VOR of all six semicircular canals of the ear labyrinth. The basic size of this search is the so-called gain - the ratio of average eye speed and head speed. The VOR generates eye movements that are equal to but opposite to the rotation of the head. Therefore, the gain of VOR in healthy people is approaching unity. Unlike the thermal test, vHIT has a very narrow range of normal values, and patients with VM tolerate vHIT well. SVV is a completely painless and short test (lasting 5 to 15 minutes) that

gives us the numerical value of the inclination of your head to examine the interaction between your body and the balance of the senses. We will examine it with an opaque plastic bucket on the bottom which will be glued to a mobile phone with an active „Visual vertical“ iPhone app. The iPhone app on the bottom of the bucket will show a straight red diametric line with a zero line at 90° corresponding to the true vertical. The participants will adjust the red line to what they believe is the position of "gravitational vertical". The test will be repeated six times and the audiologist will calculate the average value of the iPhone app results. These are new diagnostic tests in the world of vertigo that we will examine in the acute and symptom-free phases of VM.

4. Pure tone audiometry (PTA) recording:
   PTA is a key search in audiology that examines hearing thresholds for pure tones, so it is a tonal audiogram, a graphical representation of tonal audiometry, a function of intensity and frequency. By measuring tonal audiometry we can quantify and qualify hearing impairments. The search is completely non-invasive, it is performed in an acoustically insulated cabin using headphones when measuring air conductivity, or a bone vibrator when measuring bone conductivity. The time required to measure the audiometry is about 20 minutes and we will record this search only in the acute phase of VM. The mentioned examination is the standard in the approach to the subject with acute vestibular syndrome, ie spontaneous and continuous vertigo. Hearing impairment in an acute attack of VM from hearing impairment, tonal noise to a feeling of fullness in the ear can be found in 20 to 40 % of patients.
5. Application of antimigraine therapy:
   After the diagnosis is made, the subject in an acute VM attack will start symptomatic therapy. These are medicines (ketoprofen 100 mg iv. and granisetron 3 mg iv.) for the acute attack of VM that acts on the most common symptoms such as vertigo, nausea, and headache.
6. The brain MRI:
   Within 5 to 10 days of an acute VM attack, the subject will take a pre-arranged magnetic brain scan at the Clinical Department of Diagnostic and Interventional Radiology, CHC Split to rule out other central neurological disorders.

WHAT ARE THE POSSIBLE BENEFITS OF PARTICIPATION FOR RESPONDENTS?

There is no guarantee that you will benefit from participating in the research. If you agree to participate in this research, we guarantee you an individualized approach to your VM diagnosis. It involves a detailed examination of your neurological and balance system. With this research, you have a unique opportunity to test the functionality of your hearing and balance system. All this will be analyzed by a multidisciplinary scientific team consisting of neuroscientists, otorhinolaryngologists, neurologists, and radiologists. The ultimate benefit of this research is the potential detection of the triggers of your disorder. During the research, you will be provided with an insight into all new findings, as well as all relevant findings of the performed searches.

WHAT ARE THE POSSIBLE RISKS OF PARTICIPATING IN THIS RESEARCH?

• **Examination of auditory and vestibular function:** there are no significant risks and dangers when examining vestibular function by performing a *Dix Hallpike* positional test, SVV, and vHIT, as well as recording audiometry, in this study in an adult diagnosed with VM. These diagnostic tests are standardized methods for diagnosing VM that serve to distinguish VM from similar disorders. There is a possibility of transient nausea when performing the *Dix Hallpike* positional test and vHIT.

• **Prescribing antimigraine therapy for the acute VM attack**: Once the diagnosis of VM has been diagnosed, the patient will be included in standard symptomatic therapy for acute VM attack by an experienced nurse in a controlled hospital setting. We provide proven effective drugs that relieve the symptoms of vertigo and headache (granisetron 3 mg. iv. and ketoprofen 100 mg iv.) in acute VM attacks. There is a possibility of bruising at the site of placement of the needle for administering the drug to the ulnar vein.

• **MRI of the brain:** in the symptom-free phase of the VM, you should have an MRI of the brain. This diagnostic method takes 20 to 40 minutes and there is the possibility of transient noise and discomfort produced by the machine itself to record your brain. It is a non-invasive and very informative test that will be read by a radiology specialist.

MUST PARTICIPATE?

It is up to you to freely decide whether you want to participate in this research or not. If you choose to participate, you will receive this notice to sign (keep one copy). Your participation is voluntary, and you can withdraw freely and without any consequences at any time, without giving a reason. You will continue to be treated in the way that is usual for your illness. If you decide to discontinue participation, please inform the principal investigators and their collaborators on time.

CONFIDENTIALITY AND INSPECTION OF DOCUMENTATION

The introductory part of the interview will emphasize the confidentiality of information and the content of interviews between respondents and researchers. By giving informed consent, respondents sign that they are familiar with the objectives of the research and agree to participate in the research. Your data will be entered electronically in the hospital information system (BIS) when you come to the emergency ENT department with acute vertigo. Principal investigators and their collaborators will adhere to internal procedures for the protection of personal data. You will be entered into the database (excel tables) using the code name of this survey (OSCAR 1, OSCAR 2, etc.). Your medical records will be reviewed by principal investigators and their collaborators. Representatives of the Ethics Committee of the CHC Split can also have access to the documentation.

WHAT WILL THE DATA OBTAINED IN THIS SCIENTIFIC RESEARCH BE USED FOR?

Data from this scientific research can be of practical use, but also scientific, and will be published in scientific publications. Your identity in the BIS will be visible to other doctors at the CHC Split, but it will remain anonymous in our database.

WHO ORGANIZES AND FINANCES THE RESEARCH?

The examination was organized by doctors from ENT Clinic, in Split, Croatia. Our studies do not require additional funding from the CHC Split or other institutions and individuals because standardized tests will be performed for patients with VM.

WHO REVIEWED THIS TRIAL?

This study was reviewed by the Ethics Committees of the CHC Split, which approved this research after reviewing the relevant documentation. The study is conducted by all applicable guidelines, which aim to ensure the proper conduct and safety of the persons participating in this scientific research, including the Fundamentals of Good Clinical Practice and the Declaration of Helsinki.

WHOM TO CONTACT FOR FURTHER INFORMATION?

If you need additional information, feel free to contact the principal investigators.

Any respondent may at any time request the deletion of personal data from the research medical report by sending a simple request to the study coordinator, Zoran Đogaš, MD, PhD (e-mail contact: zdogas@mefst.hr). If respondents do not request the withdrawal from the study, we would like to emphasize that published, anonymized research results cannot be removed (e.g., research results are published in a scientific journal). To promote free access to scientific information and avoid unnecessary research, anonymous transcribed data from conducted examinations will be available in the OSF register (Open Science Framework, https://osf.io/). Anonymity will be ensured by removing all names.

VIOLATION OF DATA CONFIDENTIALITY

In the event of a data breach, we will contact the affected respondents and the data will be temporarily removed from the compromised storage.

INFORMED CONSENT

Participation in this study is voluntary, and respondents can withdraw from the study at any time without giving reasons for withdrawal. If you want to withdraw from the study, please contact the principal investigator, Zoran Đogaš, MD, PhD (e-mail contact: zdogas@mefst.hr).

- I have read information about the study. I had the opportunity to ask questions and get satisfactory answers. I had enough time to decide whether to participate in this study.
- I am aware that my participation in the study is voluntary. Also, I know that I can choose not to participate or withdraw from this study at any time. I am not obliged to state the reasons for non-participation or withdrawal from the study.
- I consent to the video recording of the eye movement during the oto-neurological examination.
- I consent to the collection and use of my examination data from the examination in the acute attack and the control.
- I consent to my anonymized and transcribed examination data being publicly available on the OSF scientific platform. I understand that this means that my anonymized data may be used for research purposes other than those described above. I am also aware that this means that my anonymized data may be used in countries outside Europe and that data use regulations may be different from those in the European Union.
- I understand that the responsible individuals like the main researchers and their associates, and the members of the CHC Split have access to my medical documentation. I permit these individuals to access my medical records.
- I agree that my family doctor (or family member) be aware of my participation in the above scientific research.
- I want to participate in this study.

Respondent:

Name and surname (in block letters): ____
Date: ____
Signature:____

The person who conducted the notification procedure for the respondent and the consent to participate:

Name and surname (in block letters): ____
Date: ____
Signature:____

Principal Investigator of the project:

Name and surname (in block letters): ____
Date: ____
Signature:____
