# Supplementary material for: Clinical Features, Video Head Impulse Test, and Subjective Visual Vertical of Acute and Symptom-Free Phases in Patients with Definite Vestibular Migraine
Source: Biomedicines. 2025 Mar 30;13(4):825. doi: 10.3390/biomedicines13040825 (PMC12024932; doi:10.3390/biomedicines13040825)
Supplement: Supplementary file 1 [file biomedicines-13-00825-s001.zip › Supplementary File S3 - Diagnostic criteria.pdf]

Supplementary File S3

Diagnostic criteria for patients with definite vestibular migraine[1,2]

1. At least 5 episodes of moderate or severe vestibular symptoms, which last from 5 minutes to 72 hours.
2. Migraine with or without aura in medical history, according to the International Classification Headache (ICHD).
3. One or more features of migraines with at least 50% vestibular episodes:

- headache with at least two of the following characteristics: unilateral localization, pulsation, moderate or severe pain, worsening with routine physical activities.
- photophobia or phonophobia.
- visual aura.

4. It cannot be classified as any other ICHD diagnosis.

[1] *Headache Classification Committee of the International Headache Society The International Classification of Headache Disorders. Cephalalgia. 2018; 38 (3rd edition): 1-211.*

[2] *Lempert T, Olesen J, Furman J, Waterston J, Seemungal B, Carey J, Bisdorff A, Versino M, Evers S, Newman-Toker D. Vestibular migraine: diagnostic criteria. J Vestib Res. 2012;22(4):167-72.*
