# Supplementary material for: Clinical Features, Video Head Impulse Test, and Subjective Visual Vertical of Acute and Symptom-Free Phases in Patients with Definite Vestibular Migraine
Source: Biomedicines. 2025 Mar 30;13(4):825. doi: 10.3390/biomedicines13040825 (PMC12024932; doi:10.3390/biomedicines13040825)
Supplement: Supplementary file 1 [file biomedicines-13-00825-s001.zip › Supplementary File S4 - LMM results.pdf]

Supplementary File S4

Detailed linear mixed model (LMM) results for SVV and vHIT measures

Table of Contents

1. Subjective visual vertical (SVV) 2

Table S1. Results of the linear mixed model longitudinal analysis for SVV 2

Figure S1. 3

Figure S2. 4

Figure S3. 5

2. Right ear lateral semicircular canal 6

Table S2. Results of the linear mixed model longitudinal analysis for right ear lateral semicircular canal 6

Figure S4. 7

Figure S5. 8

Figure S6. 9

3. Right ear anterior semicircular canal 10

Table S3. Results of the linear mixed model longitudinal analysis for right ear anterior semicircular canal 10

Figure S7. 11

Figure S8. 12

Figure S9. 13

4. Right ear posterior semicircular canal 14

Table S4. Results of the linear mixed model longitudinal analysis for right ear posterior semicircular canal 14

Figure S10. 15

Figure S11. 16

Figure S12. 17

5. Left ear lateral semicircular canal 18

Table S5. Results of the linear mixed model longitudinal analysis for left ear lateral semicircular canal 18

Figure S13. 19

Figure S14. 20

Figure S15. 21

6. Left ear anterior semicircular canal 22

Table S6. Results of the linear mixed model longitudinal analysis for left ear anterior semicircular canal 22
Figure S16. 23
Figure S17. 24
Figure S18. 25
7. Left ear posterior semicircular canal 26
Table S7. Results of the linear mixed model longitudinal analysis for left ear posterior semicircular canal 26
Figure S19. 27
Figure S20. 28
Figure S21. 29

A linear mixed-effects model was used to assess the effects of time (ictal vs. inter-ictal), age, and sex on subjective visual vertical (SVV) scores in patients with vestibular migraine (VM). The model included Time (Ictal vs. Inter-ictal) as a fixed effect, with age and sex (coded as 1 = male, 2 = female) as covariates. A random intercept for each participant was included to account for the correlation of repeated measures within individuals.

1. Subjective visual vertical (SVV)

Table S1. Results of the linear mixed model longitudinal analysis for SVV

Predictors	Estimate	Std. Error	df	t-value	p-value
**Fixed Effects**					
Intercept (Inter-Ictal baseline)	1.20	0.43	28.97	2.755	0.010*
Period (Ictal vs. Inter-Ictal)	0.678	0.113	30.00	5.971	$1.51 \times 10^{-6*}$
Gender (2 = Female)	-0.245	0.329	19.34	-0.744	0.463
Age	-0.0037	0.006	19.34	-0.594	0.557
**Random Effects**					
σ^2 (Residual variance)			0.2001		
τ_{00} (ID variance)			0.1947		
N (Participants)			31		
Observations			62		

τ_{00} (Tau), the variance of the random intercepts for the grouping factor (ID) representing the variability in the baseline levels between groups; σ^2, residual variance representing the within-group variability (i.e., the variability not explained by the grouping factor). Significant p-values are marked in bold.

The results indicated a significant increase in SVV deviation during ictal episodes compared to the inter-ictal baseline ($\beta = 0.678$, SE = 0.113, $p = 1.51 \times 10^{-6}$), suggesting that vestibular

function is transiently disrupted during vestibular migraine (VM) attacks. On average, SVV scores were 0.678° higher during ictal episodes, indicating a temporary impairment in spatial orientation perception associated with VM.

Additionally, neither gender ($\beta = -0.245$, SE = 0.329, $p = 0.463$) nor age ($\beta = -0.0037$, SE = 0.006, $p = 0.557$) significantly influenced SVV deviation, suggesting that SVV changes during vestibular migraine attacks occur independently of sex and age-related differences.

The random effect for participants ($\tau_{00} = 0.1947$) suggests moderate inter-individual variability in baseline (inter-ictal) SVV values, meaning that some individuals naturally have greater SVV deviations than others. In contrast, the residual variance ($\sigma^2 = 0.2001$) is relatively small, indicating that within-individual SVV variability over time is limited. This reinforces the idea that SVV fluctuations are primarily driven by the transition between ictal and inter-ictal states in VM rather than random variation within a given state.

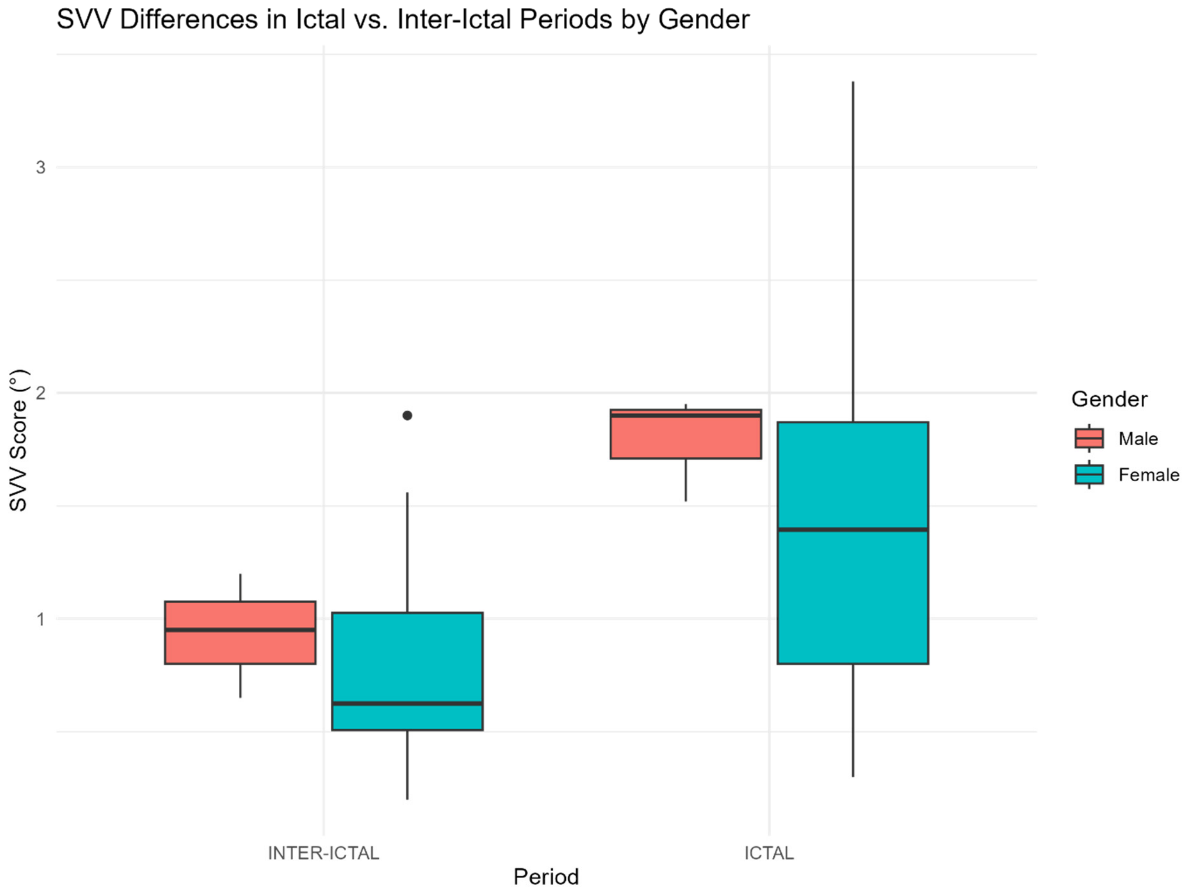

Figure S1. Boxplot showing Subjective Visual Vertical (SVV) deviations across Ictal and Inter-Ictal periods in patients with Vestibular Migraine (VM), stratified by gender. SVV scores are presented separately for males (red) and females (blue). During ictal episodes, SVV deviation increases in both genders, with greater variability observed in females. The black horizontal lines represent median values, and the boxes indicate interquartile ranges (IQR), with whiskers extending to 1.5× IQR. Outliers are shown as individual points.

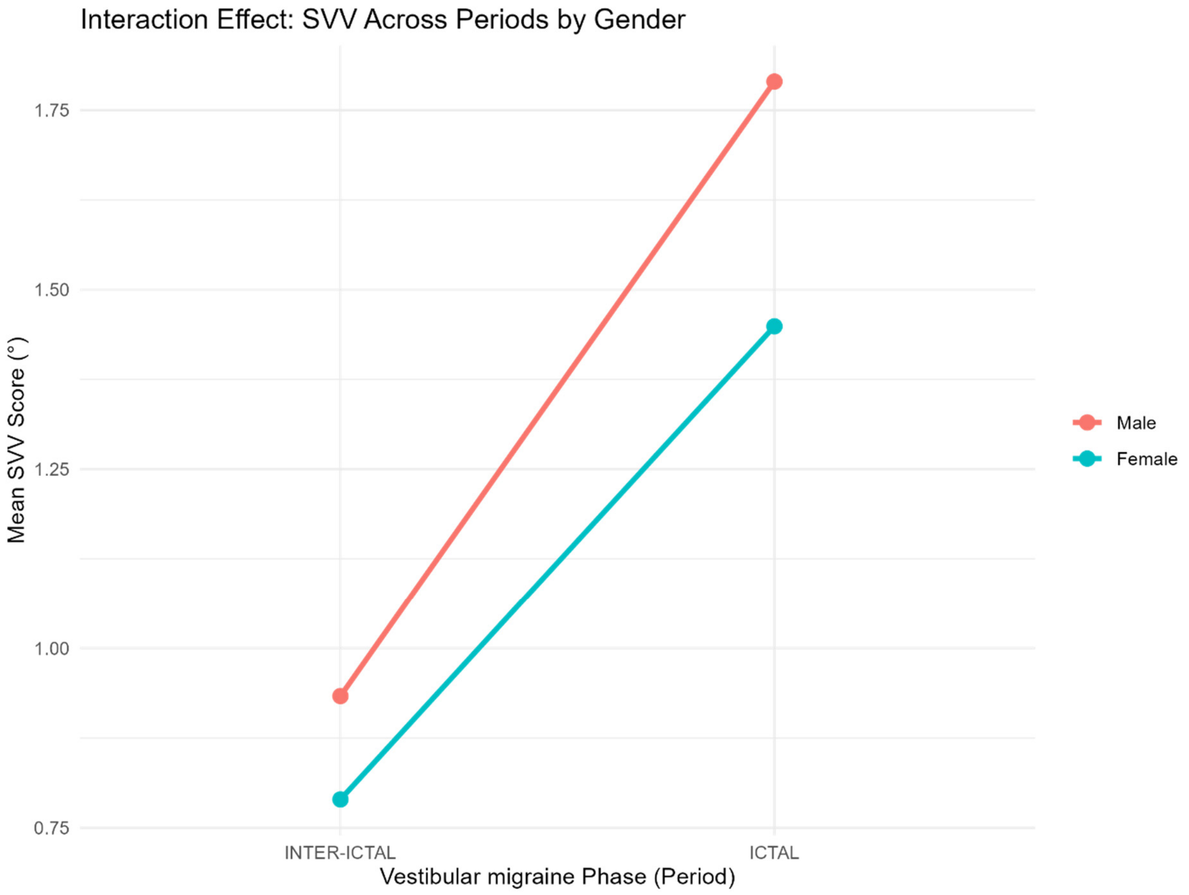

Figure S2. Interaction plot illustrating the effect of migraine phase (Ictal vs. Inter-Ictal) on Subjective Visual Vertical (SVV) scores, stratified by gender in Vestibular Migraine (VM) patients. The lines represent the mean SVV scores for males (red) and females (blue) across periods. Both genders show an increase in SVV deviation during ictal episodes, with males exhibiting a larger increase compared to females.

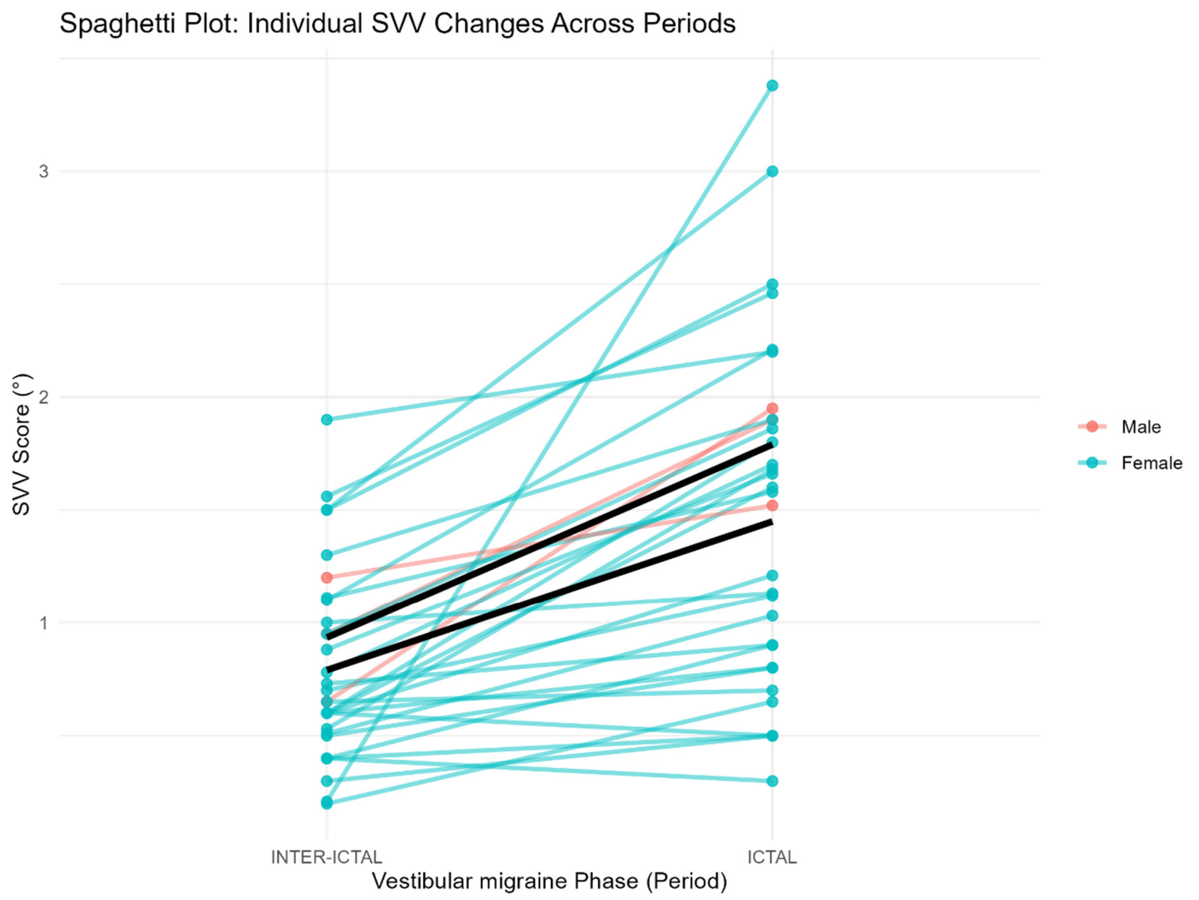

Figure S3. Spaghetti plot showing individual Subjective Visual Vertical (SVV) changes across Ictal and Inter-Ictal periods in Vestibular Migraine (VM) patients, stratified by gender. Each line represents an individual participant, illustrating within-subject variability in SVV deviation. The black lines represent the mean SVV trajectory for each gender.

A linear mixed-effects model was used to assess the effects of time (ictal vs. inter-ictal), age, and sex on each of the measured vHIT VOR gains in patients with vestibular migraine (VM). The model included Time (Ictal vs. Inter-ictal) as a fixed effect, with age and sex (coded as 1 = male, 2 = female) as covariates. A random intercept for each participant was included to account for the correlation of repeated measures within individuals.

2. Right ear lateral semicircular canal

Table S2. Results of the linear mixed model longitudinal analysis for right ear lateral semicircular canal

Predictors	Estimate	Std. Error	df	t-value	p-value
**Fixed Effects**					
Intercept (Inter-Ictal baseline)	0.9723	0.1191	27.57	8.167	7.7×10^{-9}
Period (Ictal vs. Inter-Ictal)	0.0326	0.0181	28.83	1.797	0.0831
Gender (2 = Female)	0.0674	0.0903	26.96	0.746	0.4620
Age	0.0576	0.1733	27.41	0.322	0.7501
**Random Effects**					
σ^2 (Residual variance)			0.0048		
τ_{00} (ID variance)			0.0196		
N (Participants)			30		
Observations			59		

τ_{00} (Tau), the variance of the random intercepts for the grouping factor (ID) representing the variability in the baseline levels between groups; σ^2, residual variance representing the within-group variability (i.e., the variability not explained by the grouping factor). Significant p-values are marked in bold.

The results indicate that Right Lateral semicircular canal (SC) function does not significantly differ between ictal and inter-ictal periods ($\beta = 0.0326$, SE = 0.0181, $p = 0.0831$), though a trend toward increased function during ictal episodes is observed. This suggests that Right Lateral semicircular canal gain might slightly increase during vestibular migraine (VM) attacks, but the evidence is not statistically significant ($p > 0.05$).

Additionally, neither gender ($\beta = 0.0674$, SE = 0.0903, $p = 0.4620$) nor age ($\beta = 0.0576$, SE = 0.1733, $p = 0.7501$) significantly influenced Right Lateral SC function, suggesting that variations in semicircular canal gain occur independently of sex and age-related differences in vestibular migraine patients.

The random effect for participants ($\tau_{00} = 0.0196$) suggests some inter-individual variability in baseline (inter-ictal) Right Lateral SC function, meaning that some individuals naturally have higher or lower canal function than others. In contrast, the residual variance ($\sigma^2 = 0.0048$) is small, indicating low within-individual variability over time. This suggests that the primary

fluctuations in Right Lateral SC function are related to differences between individuals rather than random fluctuations within the same person over time.

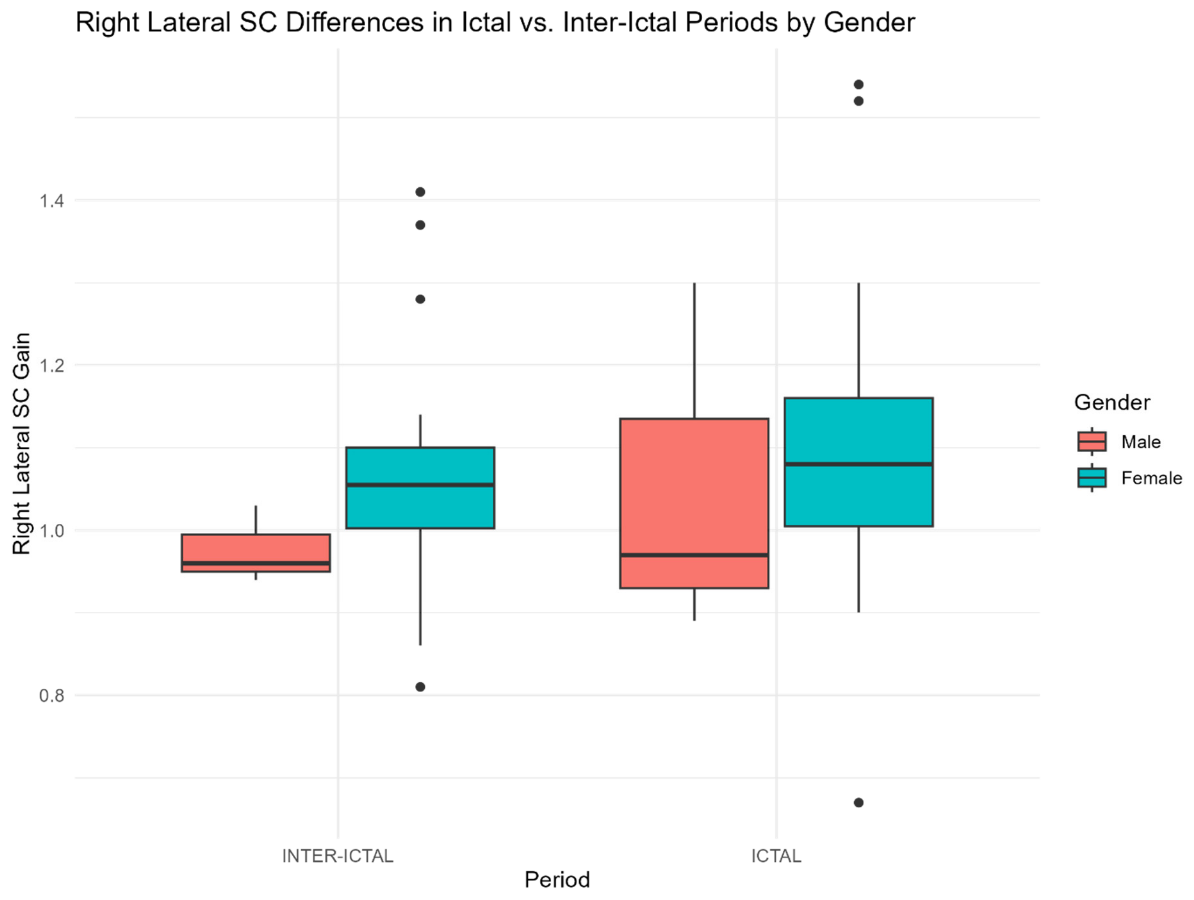

Figure S4. Boxplot showing Right Lateral Semicircular Canal (SC) gain across Ictal and Inter-Ictal periods in patients with Vestibular Migraine (VM), stratified by gender. Lateral SC gain values are presented separately for males (red) and females (blue). During ictal episodes, lateral SC function tends to increase, with greater variability observed in females. The black horizontal lines represent median values, and the boxes indicate interquartile ranges (IQR), with whiskers extending to 1.5× IQR. Outliers are shown as individual points.

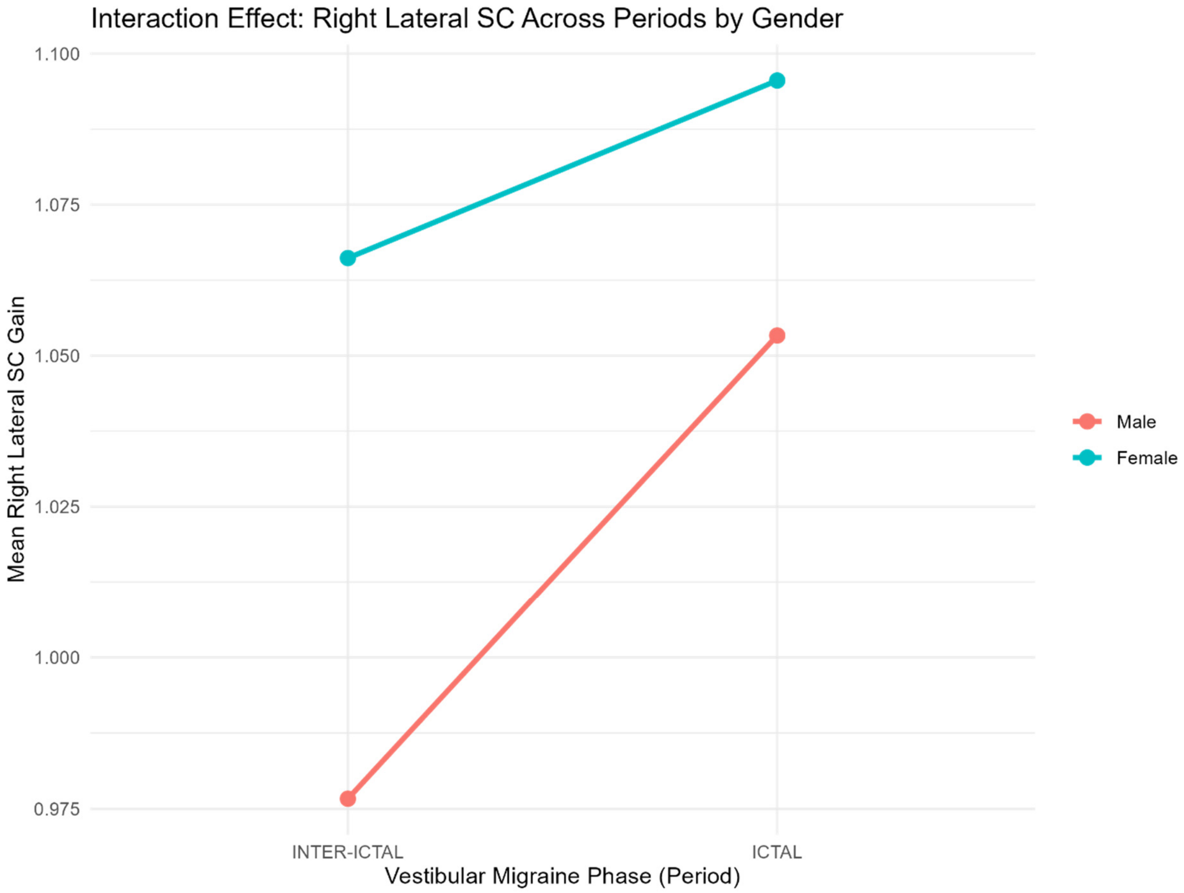

Figure S5. Interaction plot illustrating the effect of vestibular migraine phase (Ictal vs. Inter-Ictal) on Right Lateral Semicircular Canal (SC) function, stratified by gender in Vestibular Migraine (VM) patients. The lines represent the mean Right Lateral SC gain for males (red) and females (blue) across periods. Both genders show a slight increase in lateral SC gain during ictal episodes, with males exhibiting a larger increase compared to females.

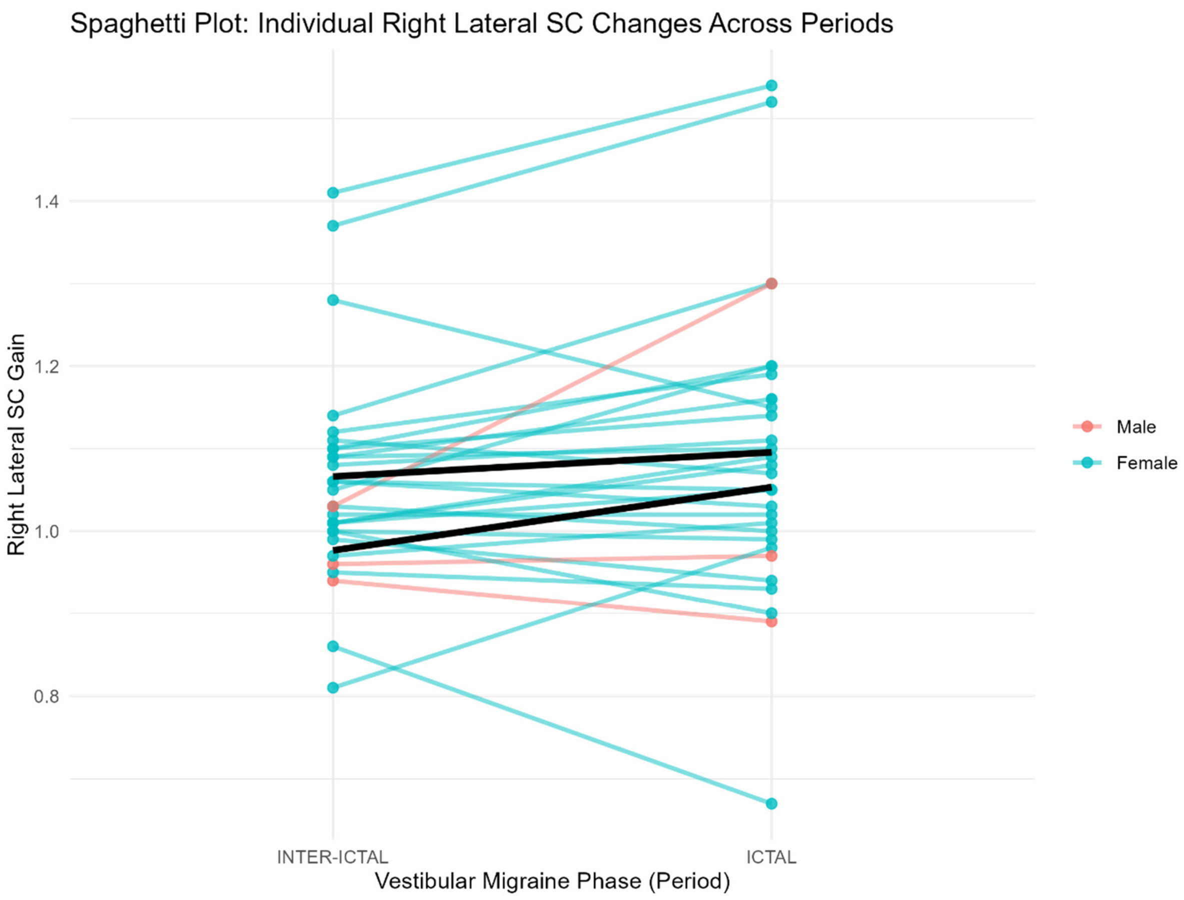

Figure S6. Spaghetti plot showing individual Right Lateral Semicircular Canal (SC) function changes across Ictal and Inter-Ictal periods in Vestibular Migraine (VM) patients, stratified by gender. Each line represents an individual participant, illustrating within-subject variability in Right Lateral SC gain. The black lines represent the mean Right Lateral SC trajectory for each gender.

3. Right ear anterior semicircular canal

Table S3. Results of the linear mixed model longitudinal analysis for right ear anterior semicircular canal

Predictors	Estimate	Std. Error	df	t-value	p-value
**Fixed Effects**					
Intercept (Inter-Ictal baseline)	0.871	0.119	29.57	7.332	3.96×10^{-8}
Period (Ictal vs. Inter-Ictal)	0.016	0.0447	28.96	0.358	0.723
Gender (2 = Female)	0.0195	0.0879	26.77	0.221	0.827
Age	0.000 16	0.001 71	28.04	0.094	0.925
**Random Effects**					
σ^2 (Residual variance)			0.0294		
τ_{00} (ID variance)			0.0061		
N (Participants)			30		
Observations			59		

τ_{00} (Tau), the variance of the random intercepts for the grouping factor (ID) representing the variability in the baseline levels between groups; σ^2, residual variance representing the within-group variability (i.e., the variability not explained by the grouping factor). Significant p-values are marked in bold.

The results indicate that Anterior Semicircular Canal (SC) function does not significantly differ between ictal and inter-ictal periods (β = 0.016, SE = 0.0447, p = 0.723). This suggests that the function of the anterior SC remains stable during vestibular migraine (VM) attacks, with no strong evidence of phase-dependent changes.

Additionally, neither gender (β = 0.0195, SE = 0.0879, p = 0.827) nor age (β = 0.00016, SE = 0.00171, p = 0.925) significantly influenced Anterior SC function, indicating that variations in semicircular canal gain occur independently of sex and age-related differences in VM patients.

The random effect for participants (τ_{00} = 0.0061) suggests some inter-individual variability in baseline (inter-ictal) Anterior SC function, meaning that some individuals naturally have higher or lower canal function than others. In contrast, the residual variance (σ^2 = 0.0294) is small, indicating low within-individual variability over time. This reinforces the idea that the primary fluctuations in Anterior SC function are due to differences between individuals rather than random fluctuations within the same person over time.

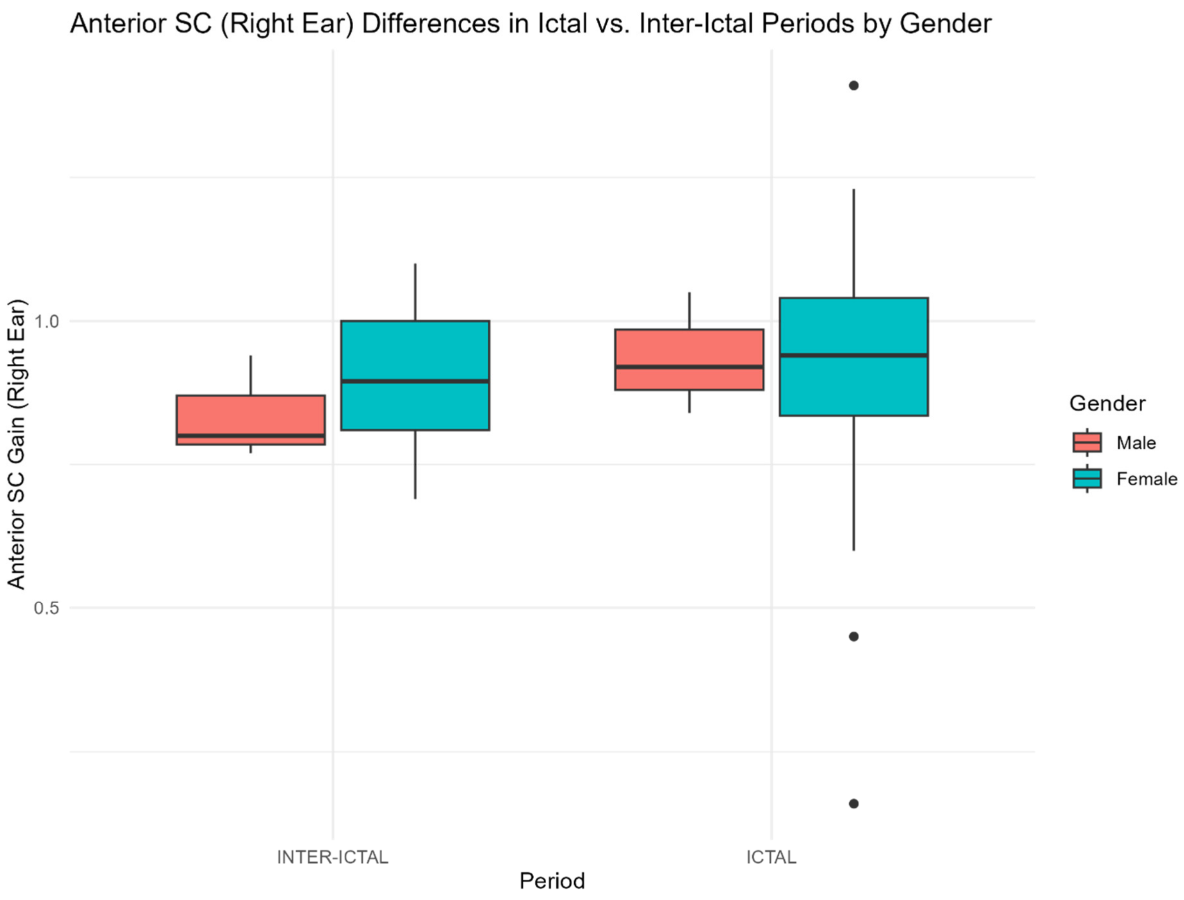

Figure S7. Boxplot showing Right Anterior Semicircular Canal (SC) gain across Ictal and Inter-Ictal periods in patients with Vestibular Migraine (VM), stratified by gender. Anterior SC gain values are presented separately for males (red) and females (blue). During ictal episodes, anterior SC function tends to increase, with greater variability observed in females. The black horizontal lines represent median values, and the boxes indicate interquartile ranges (IQR), with whiskers extending to 1.5× IQR. Outliers are shown as individual points.

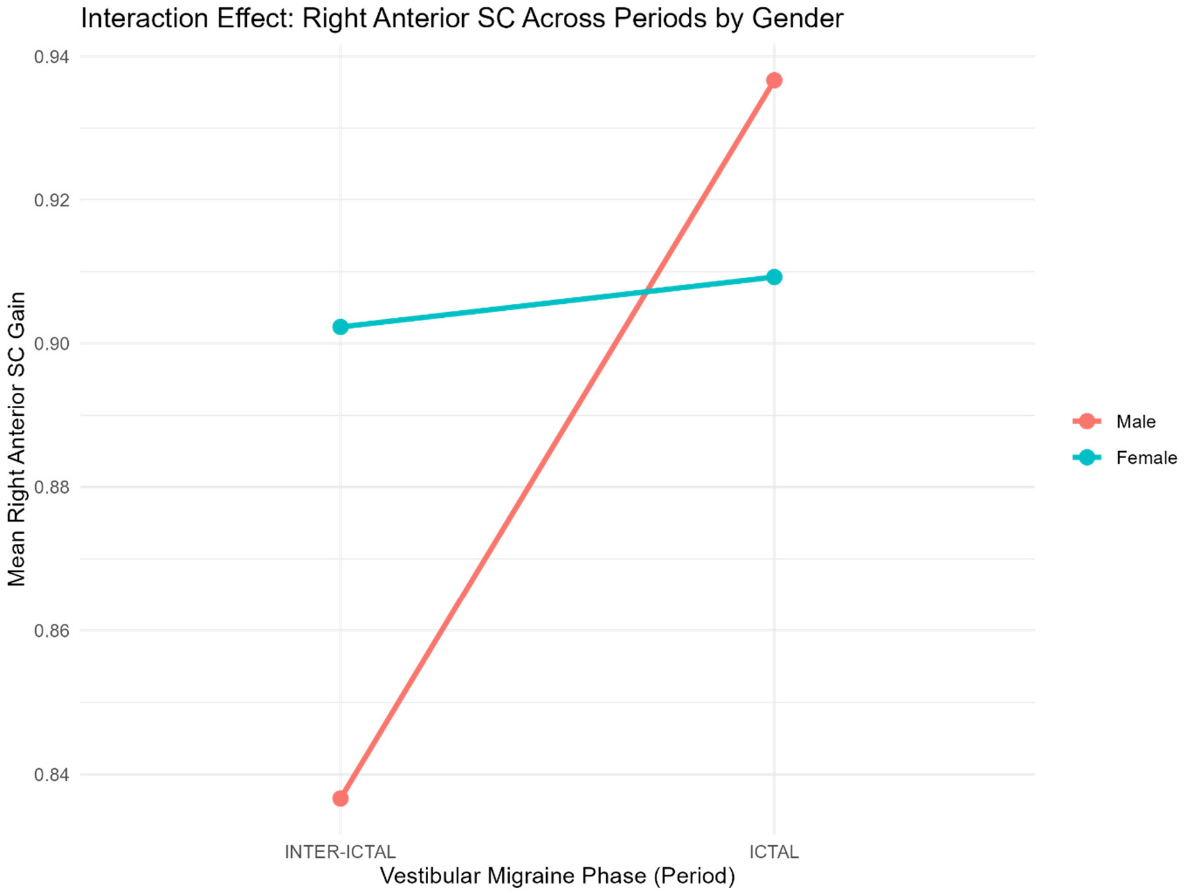

Figure S8. Interaction plot illustrating the effect of vestibular migraine phase (Ictal vs. Inter-Ictal) on Right Anterior Semicircular Canal (SC) function, stratified by gender in Vestibular Migraine (VM) patients. The lines represent the mean Right Anterior SC gain for males (red) and females (blue) across periods. Both genders show an increase in anterior SC gain during ictal episodes, with males exhibiting a larger increase compared to females. The interaction between gender and phase (Ictal vs. Inter-Ictal) was not significant.

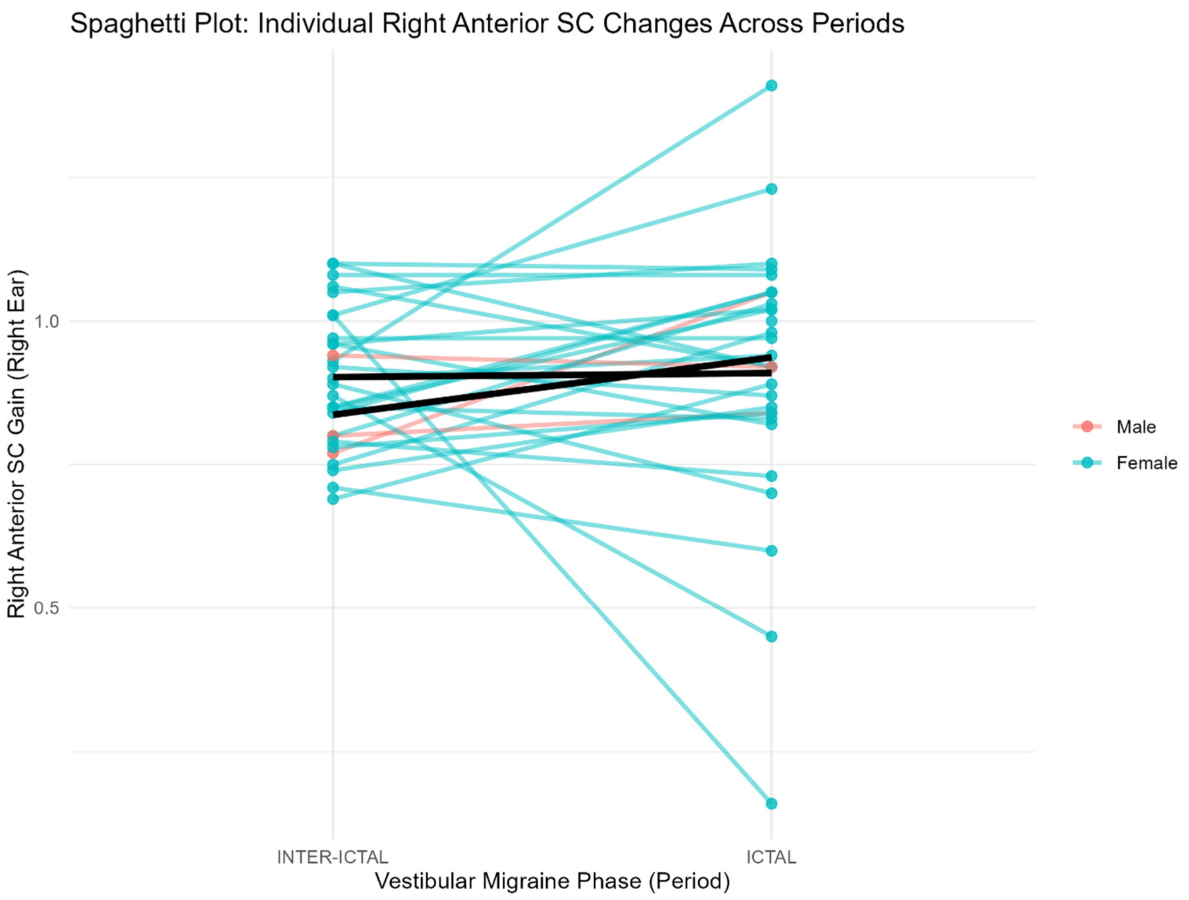

Figure S9. Spaghetti plot showing individual Right Anterior Semicircular Canal (SC) function changes across Ictal and Inter-Ictal periods in Vestibular Migraine (VM) patients, stratified by gender. Each line represents an individual participant, illustrating within-subject variability in Right Anterior SC gain. The black lines represent the mean Right Anterior SC trajectory for each gender.

4. Right ear posterior semicircular canal

Table S4. Results of the linear mixed model longitudinal analysis for right ear posterior semicircular canal

Predictors	Estimate	Std. Error	df	t-value	p-value
**Fixed Effects**					
Intercept (Inter-Ictal baseline)	0.9234	0.0991	28.90	9.315	3.33×10^{-10}
Period (Ictal vs. Inter-Ictal)	−0.0252	0.0305	28.81	−0.826	0.416
Gender (2 = Female)	0.0495	0.0740	26.86	0.669	0.509
Age	−0.0021	0.0014	27.98	−1.460	0.155
**Random Effects**					
σ^2 (Residual variance)			0.0137		
τ_{00} (ID variance)			0.0079		
N (Participants)			30		
Observations			59		

τ_{00} (Tau), the variance of the random intercepts for the grouping factor (ID) representing the variability in the baseline levels between groups; σ^2, residual variance representing the within-group variability (i.e., the variability not explained by the grouping factor). Significant p-values are marked in bold.

The results indicate that Right Posterior Semicircular Canal (SC) function does not significantly differ between ictal and inter-ictal periods (β = -0.0252, SE = 0.0305, p = 0.416). This suggests that the function of the posterior SC remains stable during vestibular migraine (VM) attacks, with no strong evidence of phase-dependent changes.

Additionally, neither gender (β = 0.0495, SE = 0.0740, p = 0.509) nor age (β = -0.0021, SE = 0.0014, p = 0.155) significantly influenced right ear Posterior SC function, indicating that variations in semicircular canal gain occur independently of sex and age-related differences in VM patients.

The random effect for participants (τ_{00} = 0.0079) suggests some inter-individual variability in baseline (inter-ictal) Right Posterior SC function, meaning that some individuals naturally have higher or lower canal function than others. In contrast, the residual variance (σ^2 = 0.0137) is small, indicating low within-individual variability over time.

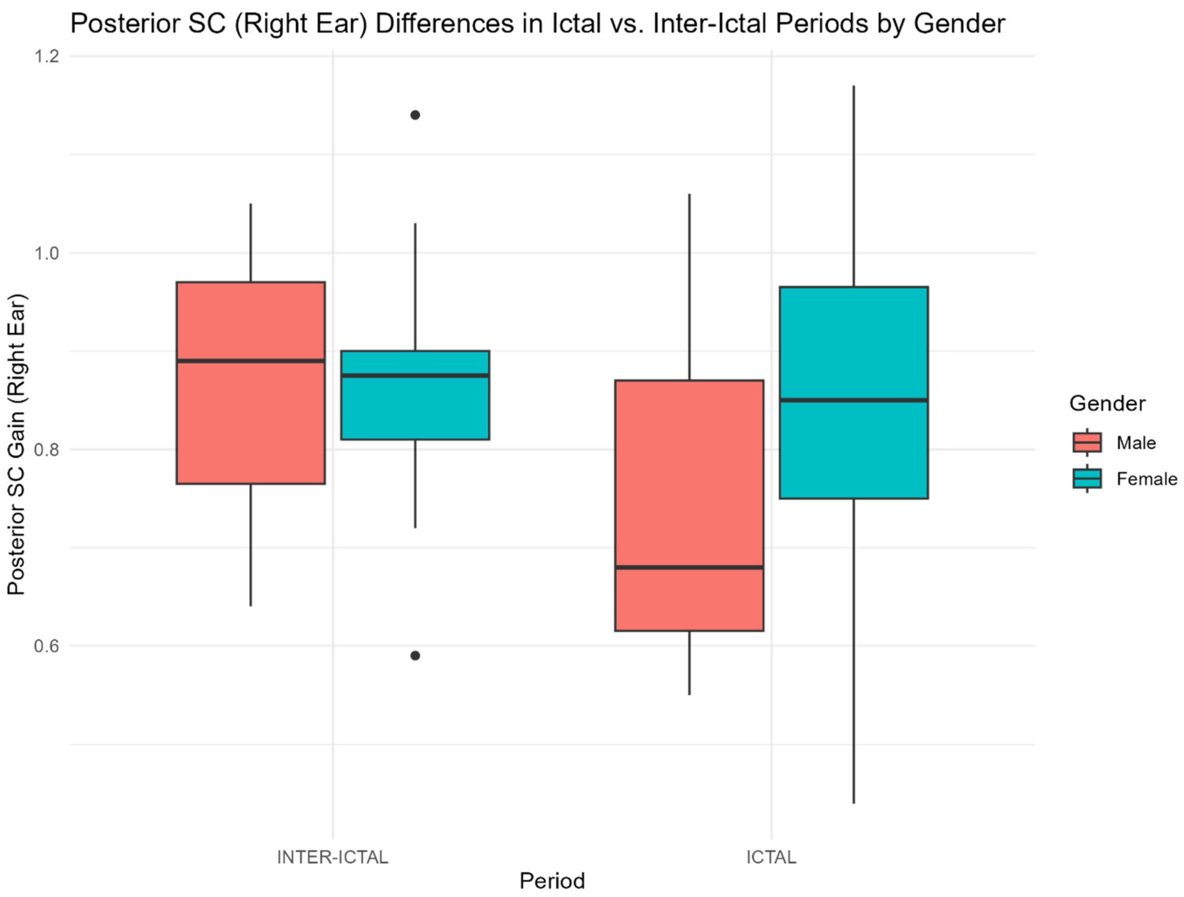

Figure S10. Boxplot showing Right Posterior Semicircular Canal (SC) gain across Ictal and Inter-Ictal periods in patients with Vestibular Migraine (VM), stratified by gender. Posterior SC gain values are presented separately for males (red) and females (blue). During ictal episodes, posterior SC function does not show a significant change, with similar variability observed in both genders. The black horizontal lines represent median values, and the boxes indicate interquartile ranges (IQR), with whiskers extending to 1.5× IQR. Outliers are shown as individual points.

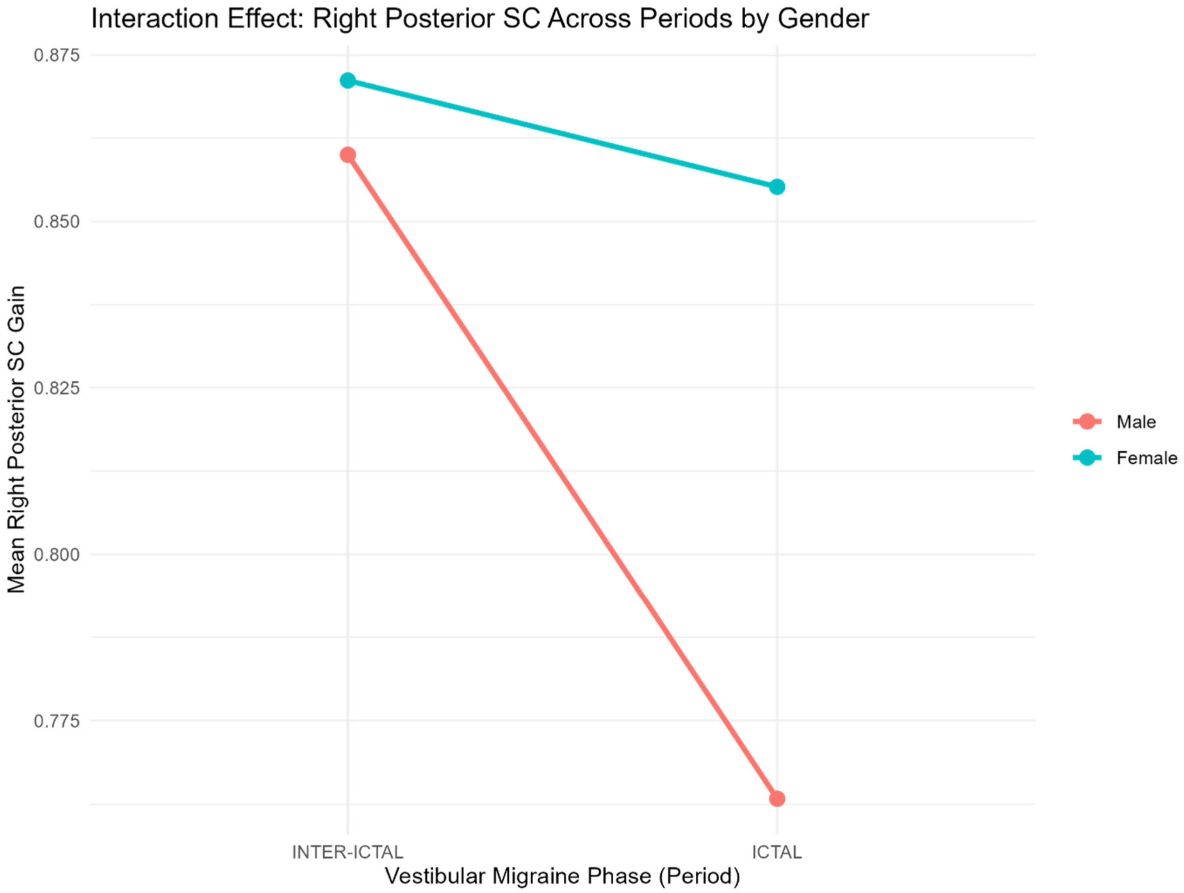

Figure S11. Interaction plot illustrating the effect of vestibular migraine phase (Ictal vs. Inter-Ictal) on Right Posterior Semicircular Canal (SC) function, stratified by gender in Vestibular Migraine (VM) patients. The lines represent the mean Right Posterior SC gain for males (red) and females (blue) across phases. Both genders exhibit stable posterior SC gain, with no significant changes observed during ictal episodes. The interaction between gender and migraine phase (Ictal vs. Inter-Ictal) was not significant.

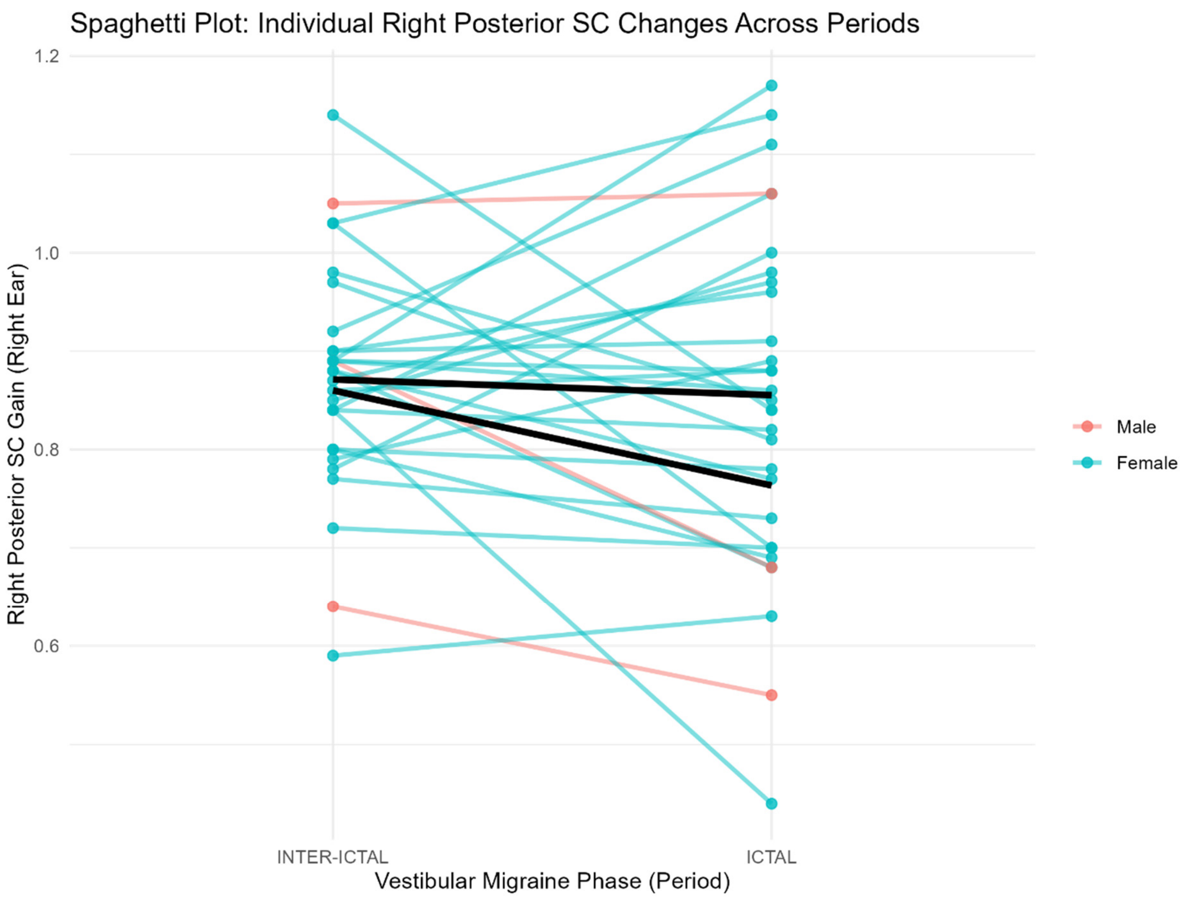

Figure S12. Spaghetti plot showing individual Right Posterior Semicircular Canal (SC) function changes across Ictal and Inter-Ictal periods in Vestibular Migraine (VM) patients, stratified by gender. Each line represents an individual participant, illustrating within-subject variability in Right Posterior SC gain. The black lines represent the mean Right Posterior SC trajectory for each gender.

5. Left ear lateral semicircular canal

Table S5. Results of the linear mixed model longitudinal analysis for left ear lateral semicircular canal

Predictors	Estimate	Std. Error	df	t-value	p-value
**Fixed Effects**					
Intercept (Inter-Ictal baseline)	0.9764	0.1326	28.57	7.364	4.5×10^{-8}
Period (Ictal vs. Inter-Ictal)	−0.0038	0.0351	28.74	−0.108	0.915
Gender (2 = Female)	0.0537	0.0995	26.96	0.539	0.594
Age	−0.0002	0.0019	27.97	−0.122	0.904
**Random Effects**					
σ^2 (Residual variance)		0.0182			
τ_{00} (ID variance)		0.0176			
N (Participants)		30			
Observations		59			

τ_{00} (Tau), the variance of the random intercepts for the grouping factor (ID) representing the variability in the baseline levels between groups; σ^2, residual variance representing the within-group variability (i.e., the variability not explained by the grouping factor). Significant p-values are marked in bold.

The results indicate that Left Lateral Semicircular Canal (SC) function does not significantly differ between ictal and inter-ictal periods (β = -0.0038, SE = 0.0351, p = 0.915). This suggests that the function of the Left Lateral SC remains stable during vestibular migraine (VM) attacks, with no strong evidence of phase-dependent changes.

Additionally, neither gender (β = 0.0537, SE = 0.0995, p = 0.594) nor age (β = -0.0002, SE = 0.0019, p = 0.904) significantly influenced Left Lateral SC function, indicating that variations in semicircular canal gain occur independently of sex and age-related differences in VM patients.

The random effect for participants (τ_{00} = 0.0176) suggests some inter-individual variability in baseline (inter-ictal) Left Lateral SC function, meaning that some individuals naturally have higher or lower canal function than others. In contrast, the residual variance (σ^2 = 0.0182) is small, indicating low within-individual variability over time.

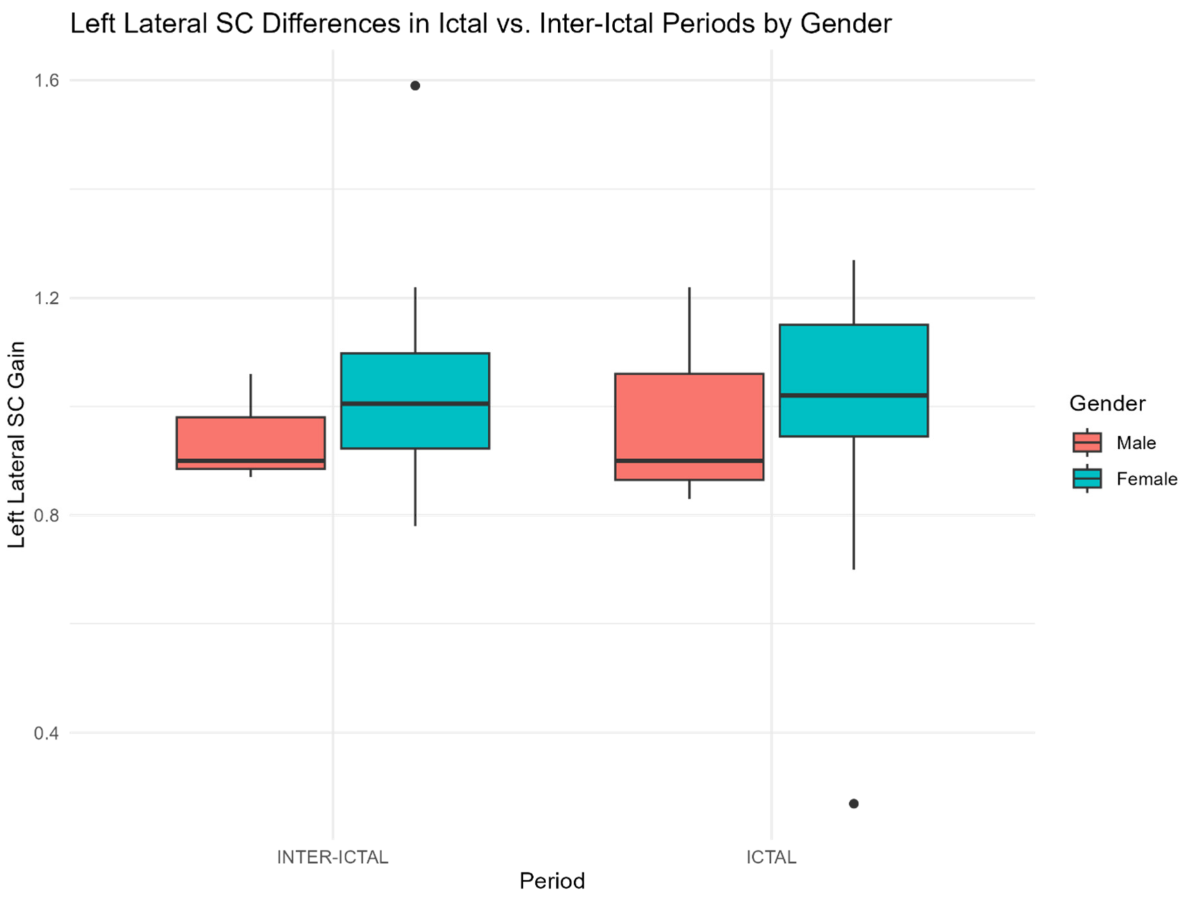

Figure S13. Boxplot showing Left Lateral Semicircular Canal (SC) gain across Ictal and Inter-Ictal periods in patients with Vestibular Migraine (VM), stratified by gender. Left Lateral SC gain values are presented separately for males (red) and females (blue). During ictal episodes, Left Lateral SC function remains stable, with no significant change observed between phases. Variability appears similar across genders. The black horizontal lines represent median values, and the boxes indicate interquartile ranges (IQR), with whiskers extending to 1.5× IQR. Outliers are shown as individual points.

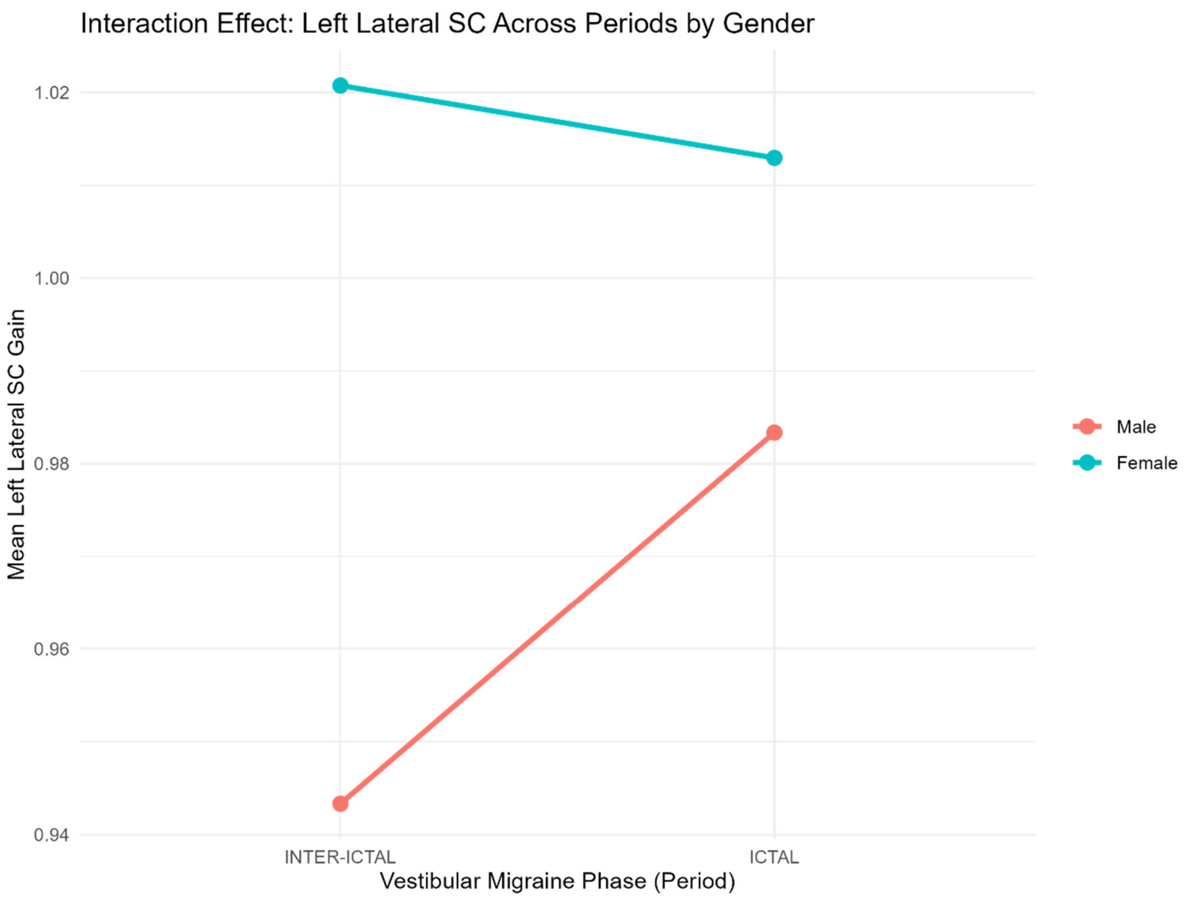

Figure S14. Interaction plot illustrating the effect of vestibular migraine phase (Ictal vs. Inter-Ictal) on Left Lateral Semicircular Canal (SC) function, stratified by gender in Vestibular Migraine (VM) patients. The lines represent the mean Left Lateral SC gain for males (red) and females (blue) across phases. Both genders exhibit stable Left Lateral SC gain, with no significant changes observed during ictal episodes. The interaction between gender and migraine phase (Ictal vs. Inter-Ictal) was not significant.

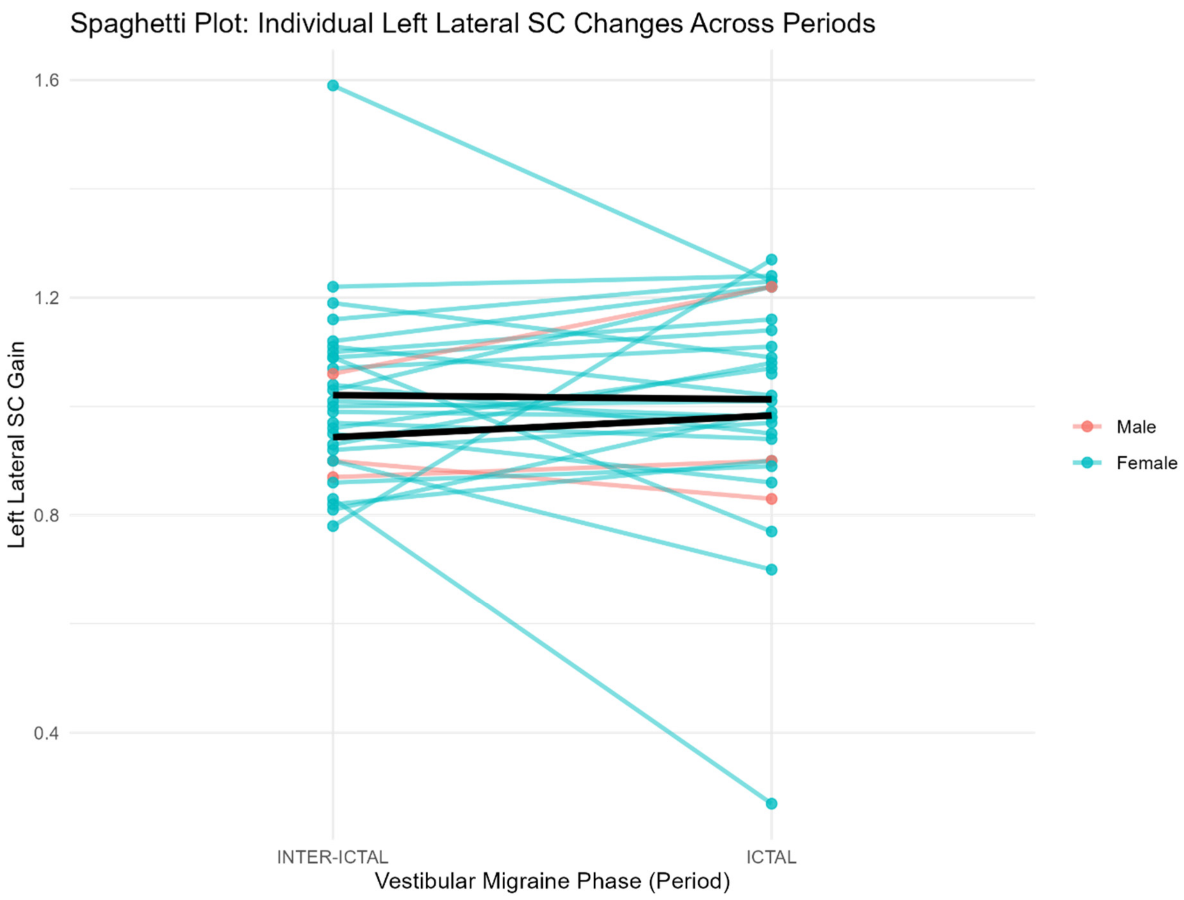

Figure S15. Spaghetti plot illustrating individual Left Lateral Semicircular Canal (SC) function changes across Ictal and Inter-Ictal periods in Vestibular Migraine (VM) patients, stratified by gender. Each line represents an individual participant, highlighting within-subject variability in Left Lateral SC gain. The black lines represent the mean Left Lateral SC trajectory for each gender. No significant phase-dependent changes were observed, suggesting stable left lateral SC function across migraine phases.

6. Left ear anterior semicircular canal

Table S6. Results of the linear mixed model longitudinal analysis for left ear anterior semicircular canal

Predictors	Estimate	Std. Error	df	t-value	p-value
**Fixed Effects**					
Intercept (Inter-Ictal baseline)	0.7812	0.1294	28.62	6.037	1.52×10^{-6}
Period (Ictal vs. Inter-Ictal)	-1.027×10^{-2}	0.0349	28.87	−0.295	0.770
Gender (2 = Female)	0.0753	0.0971	26.69	0.775	0.445
Age	-5.819×10^{-5}	0.001 88	27.80	−0.031	0.975
**Random Effects**					
σ^2 (Residual variance)		0.0178			
τ_{00} (ID variance)		0.0165			
N (Participants)		30			
Observations		59			

τ_{00} (Tau), the variance of the random intercepts for the grouping factor (ID) representing the variability in the baseline levels between groups; σ^2, residual variance representing the within-group variability (i.e., the variability not explained by the grouping factor). Significant p-values are marked in bold.

The results indicate that Left Anterior Semicircular Canal (SC) function does not significantly differ between ictal and inter-ictal periods (β = -0.0103, SE = 0.0349, p = 0.770). This suggests that Left Anterior SC function remains stable during vestibular migraine (VM) attacks, with no strong evidence of phase-dependent changes.

Additionally, neither gender (β = 0.0753, SE = 0.0971, p = 0.445) nor age (β = -0.0000582, SE = 0.00188, p = 0.975) significantly influenced Left Anterior SC function. This suggests that variations in semicircular canal gain occur independently of sex and age-related differences in VM patients.

The random effect for participants (τ_{00} = 0.0165) suggests moderate inter-individual variability in baseline (inter-ictal) Left Anterior SC function, meaning that some individuals naturally have higher or lower canal function than others. In contrast, the residual variance (σ^2 = 0.0178) is relatively small, indicating low within-individual variability over time.

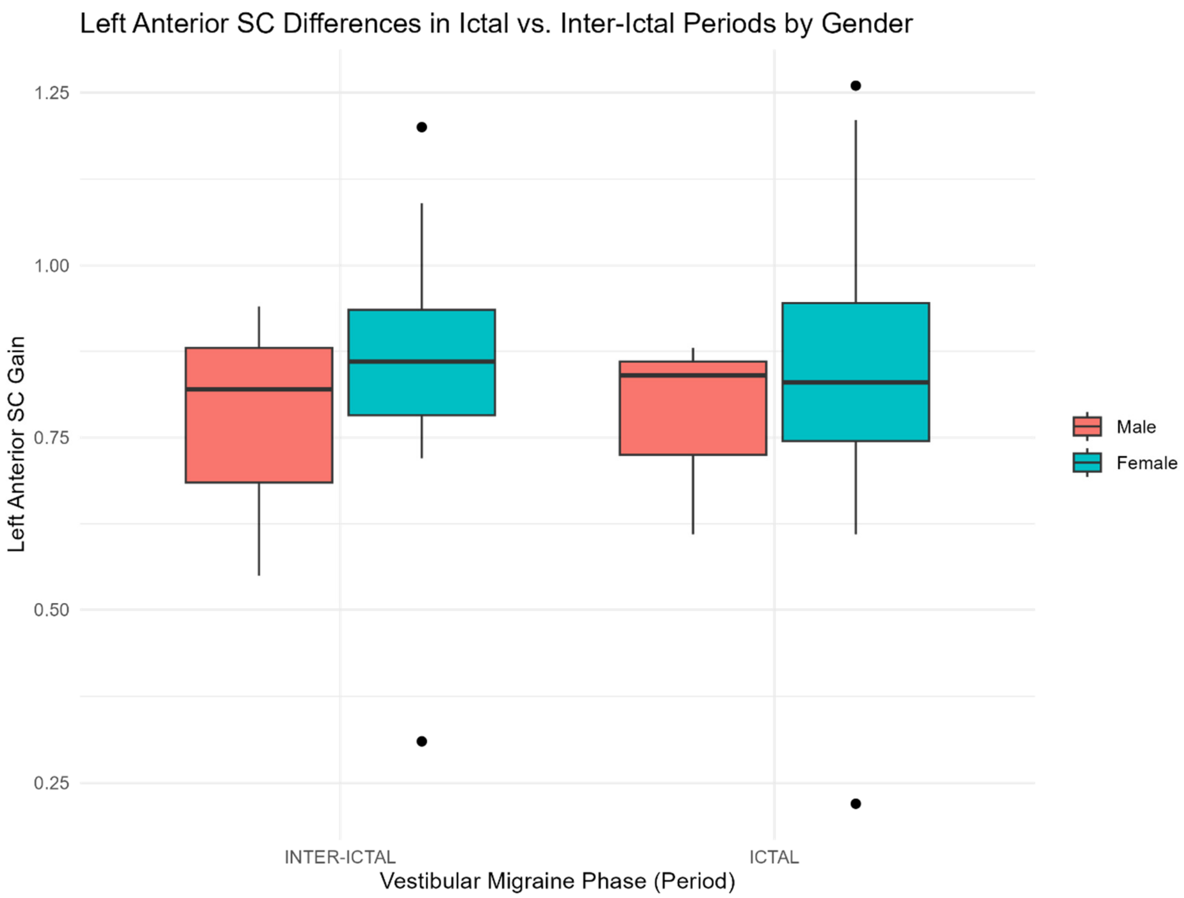

Figure S16. Boxplot illustrating Left Anterior Semicircular Canal (SC) gain across Ictal and Inter-Ictal periods in patients with Vestibular Migraine (VM), stratified by gender. Left Anterior SC gain values are displayed separately for males (red) and females (blue). No significant changes in Left Anterior SC function were observed between ictal and inter-ictal phases, indicating stability in canal function during VM episodes. Variability appears similar across genders. The black horizontal lines represent median values, while the boxes indicate interquartile ranges (IQR), with whiskers extending to 1.5× IQR. Outliers are displayed as individual points.

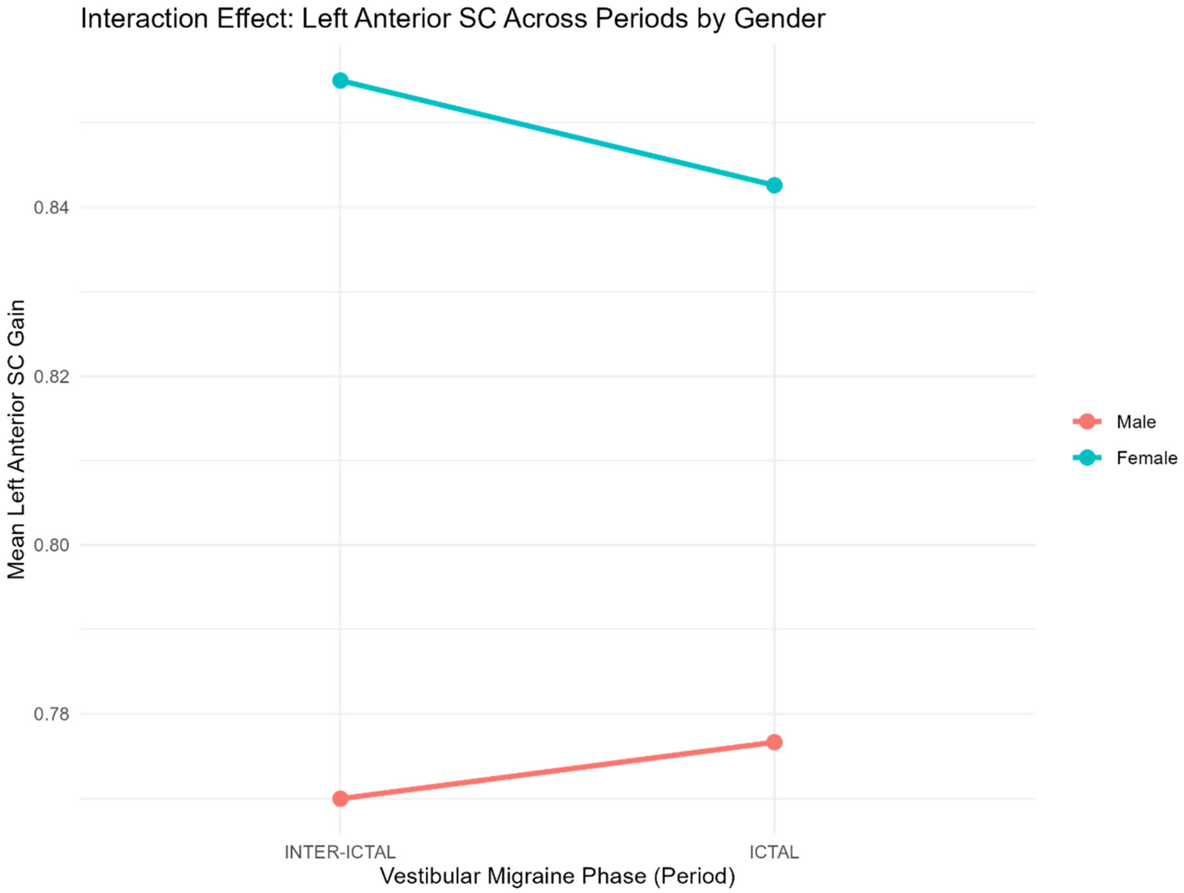

Figure S17. Interaction plot illustrating the effect of vestibular migraine phase (Ictal vs. Inter-Ictal) on Left Anterior Semicircular Canal (SC) function, stratified by gender in Vestibular Migraine (VM) patients. The lines represent the mean Left Anterior SC gain for males (red) and females (blue) across phases. Both genders exhibit stable Left Anterior SC gain, with no significant changes observed during ictal episodes. The interaction between gender and migraine phase (Ictal vs. Inter-Ictal) was not significant.

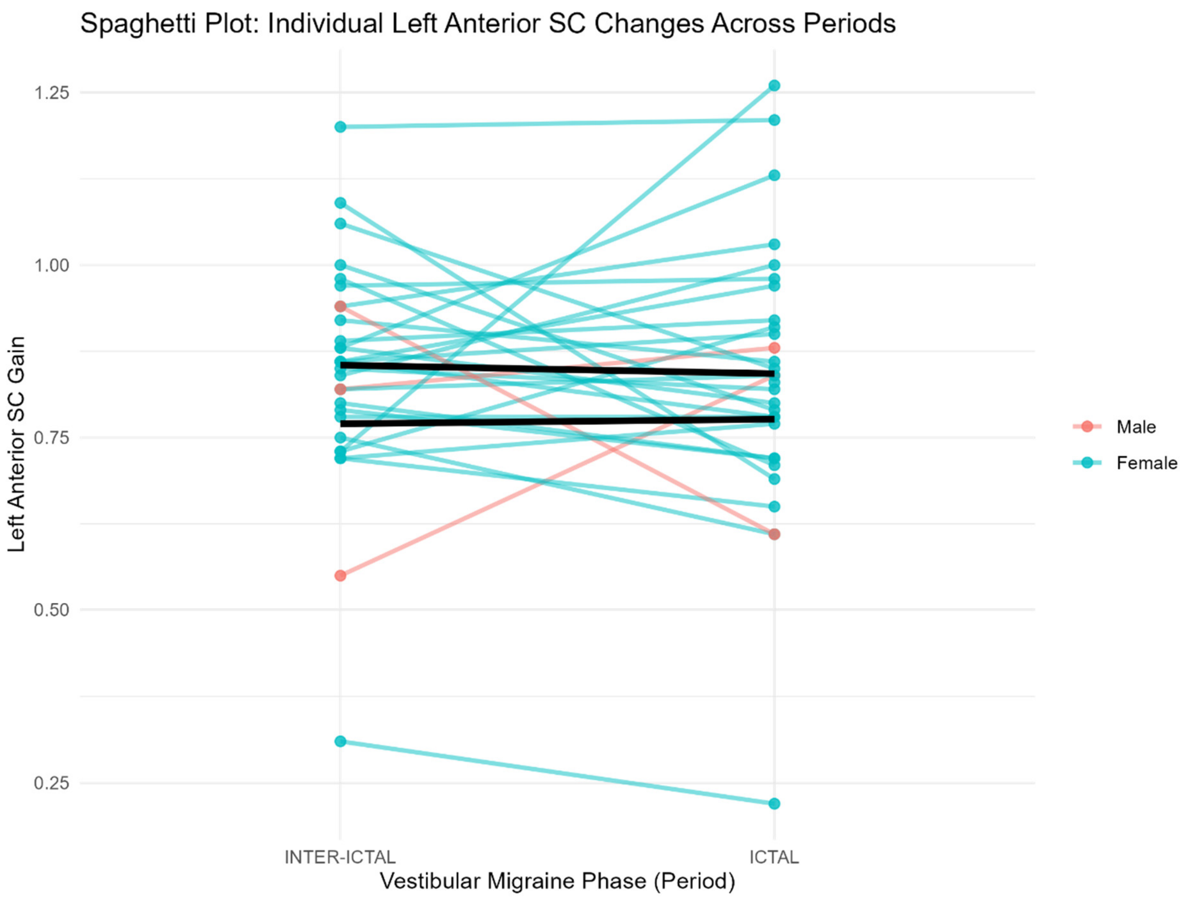

Figure S18. Spaghetti plot illustrating individual Left Anterior Semicircular Canal (SC) function changes across Ictal and Inter-Ictal periods in Vestibular Migraine (VM) patients, stratified by gender. Each line represents an individual participant, highlighting within-subject variability in Left Anterior SC gain. The black lines represent the mean Left Anterior SC trajectory for each gender. No significant phase-dependent changes were observed, suggesting stable left anterior SC function across migraine phases.

7. Left ear posterior semicircular canal

Table S7. Results of the linear mixed model longitudinal analysis for left ear posterior semicircular canal

Predictors	Estimate	Std. Error	df	t-value	p-value
**Fixed Effects**					
Intercept (Inter-Ictal baseline)	0.9167	0.1027	55.00	8.927	2.77×10^{-12}
Period (Ictal vs. Inter-Ictal)	0.0159	0.0455	55.00	0.349	0.729
Gender (2 = Female)	0.0443	0.0752	55.00	0.588	0.559
Age	−0.0014	0.0015	55.00	−0.929	0.357
**Random Effects**					
σ^2 (Residual variance)			0.0031		
τ_{00} (ID variance)			0.0000		
N (Participants)			30		
Observations			59		

τ_{00} (Tau), the variance of the random intercepts for the grouping factor (ID) representing the variability in the baseline levels between groups; σ^2, residual variance representing the within-group variability (i.e., the variability not explained by the grouping factor). Significant p-values are marked in bold.

The results indicate that Left Posterior Semicircular Canal (SC) function does not significantly differ between ictal and inter-ictal periods ($\beta = 0.0159$, SE = 0.0455, $p = 0.729$). This suggests that Left Posterior SC function remains stable during vestibular migraine (VM) attacks, with no strong evidence of phase-dependent changes.

Additionally, neither gender ($\beta = 0.0443$, SE = 0.0752, $p = 0.559$) nor age ($\beta = -0.0014$, SE = 0.0015, $p = 0.357$) significantly influenced Left Posterior SC function. This suggests that variations in semicircular canal gain occur independently of sex and age-related differences in VM patients.

The random effect for participants ($\tau_{00} = 0.0000$) suggests minimal inter-individual variability in baseline (inter-ictal) Left Posterior SC function, meaning that most individuals exhibit similar canal function. In contrast, the residual variance ($\sigma^2 = 0.0031$) is relatively small, indicating low within-individual variability over time.

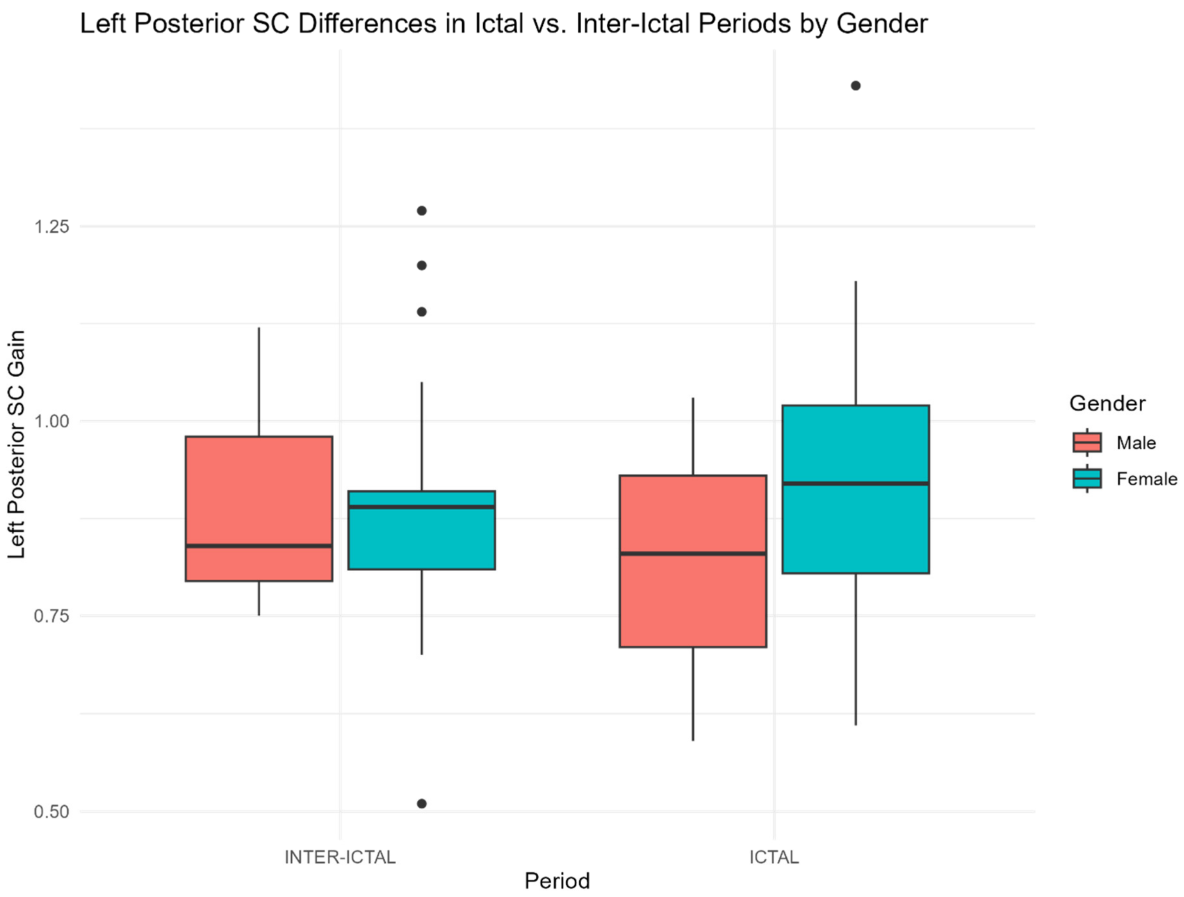

Figure S19. Boxplot illustrating Left Posterior Semicircular Canal (SC) gain across Ictal and Inter-Ictal periods in patients with Vestibular Migraine (VM), stratified by gender. Left Posterior SC gain values are displayed separately for males (red) and females (blue). No significant changes in Left Posterior SC function were observed between ictal and inter-ictal phases, indicating stability in canal function during VM episodes. Variability appears similar across genders. The black horizontal lines represent median values, while the boxes indicate interquartile ranges (IQR), with whiskers extending to 1.5× IQR. Outliers are displayed as individual points.

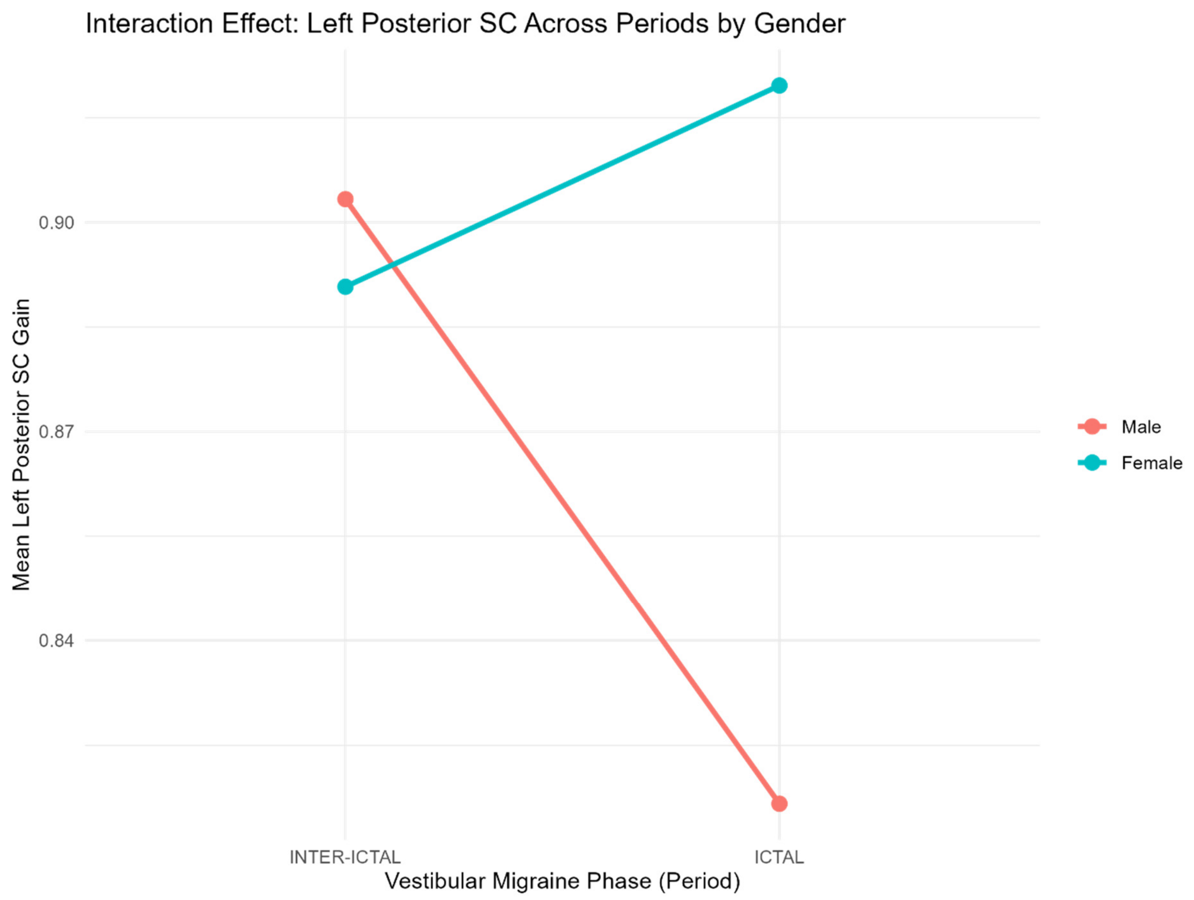

Figure S20. Interaction plot illustrating the effect of vestibular migraine phase (Ictal vs. Inter-Ictal) on Left Posterior Semicircular Canal (SC) function, stratified by gender in Vestibular Migraine (VM) patients. The lines represent the mean Left Posterior SC gain for males (red) and females (blue) across phases. Both genders exhibit stable Left Posterior SC gain, with no significant changes observed during ictal episodes. The interaction between gender and migraine phase (Ictal vs. Inter-Ictal) was not significant.

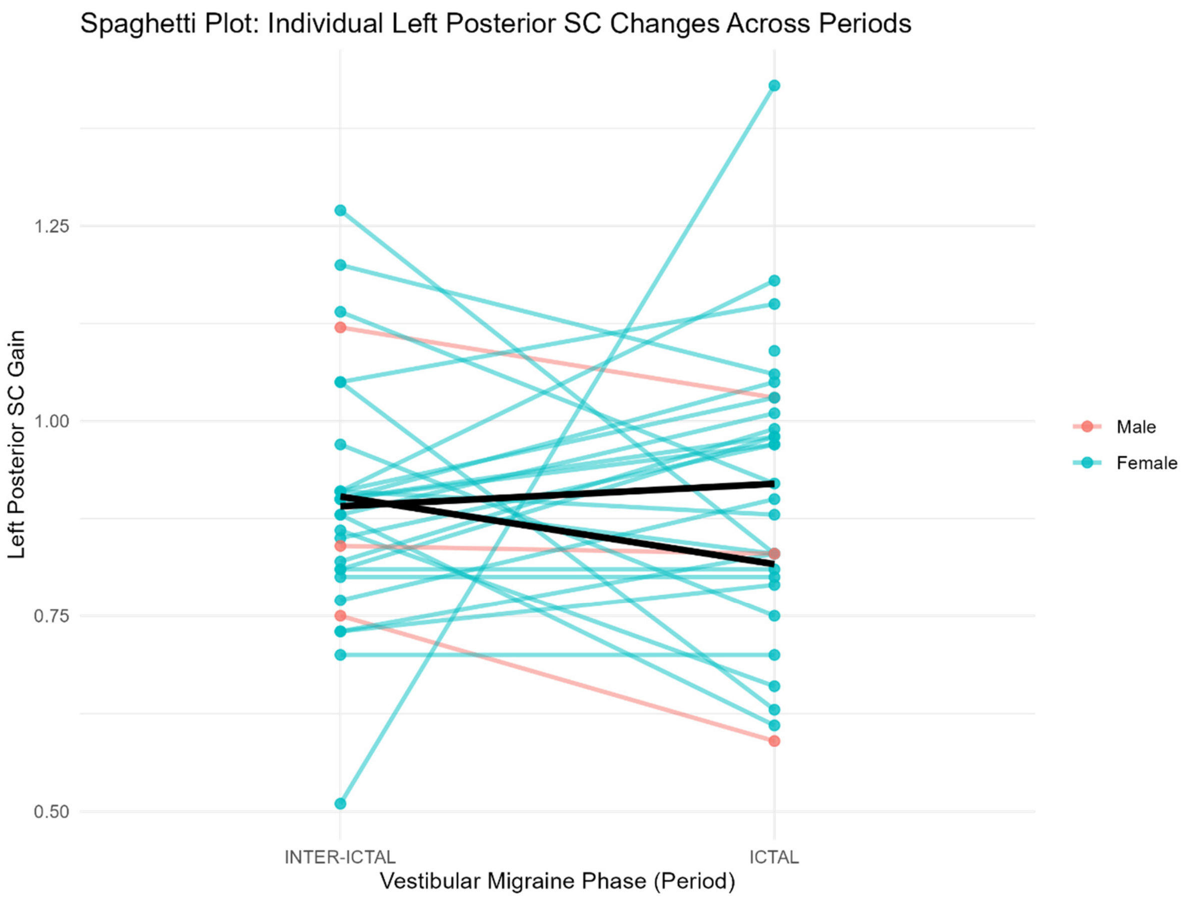

Figure S21. Spaghetti plot illustrating individual Left Posterior Semicircular Canal (SC) function changes across Ictal and Inter-Ictal periods in Vestibular Migraine (VM) patients, stratified by gender. Each line represents an individual participant, highlighting within-subject variability in Left Posterior SC gain. The black lines represent the mean Left Posterior SC trajectory for each gender. No significant phase-dependent changes were observed, suggesting stable Left Posterior SC function across migraine phases.
